# Synthesis of aryl 2-bromo-2-chloro-1,1-difluoroethyl ethers through the base-mediated reaction between phenols and halothane

**DOI:** 10.3762/bjoc.17.9

**Published:** 2021-01-11

**Authors:** Yukiko Karuo, Ayaka Kametani, Atsushi Tarui, Kazuyuki Sato, Kentaro Kawai, Masaaki Omote

**Affiliations:** 1 Faculty of Pharmaceutical Sciences, Setsunan University, 45-1, Nagaotoge-cho, Hirakata, Osaka 573-0101, Japan

**Keywords:** aryl 1,1-difluoroethyl ether, 1,1-difluoroethene, fluorine compound, halothane, phenol

## Abstract

An efficient and convenient method for the synthesis of structurally unique and highly functionalized aryl 2-bromo-2-chloro-1,1-difluoroethyl ethers has been developed. This approach exhibits a broad reaction scope, a simple operation and without the need of any expensive transition-metal catalyst, highly toxic or corrosive reagents. Notably, we demonstrate the potential utility of halothane for the synthesis of aryl *gem*-difluoroalkyl ethers containing the bromochloromethyl group.

## Introduction

**Figure 1:**
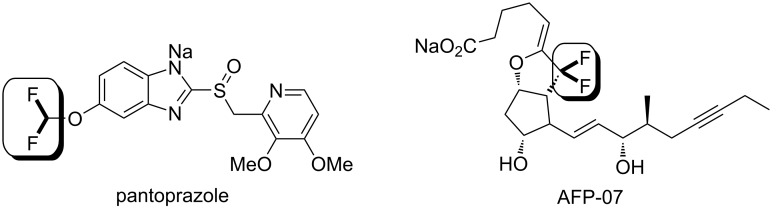
Medical compounds having a difluoromethyl group.

**Scheme 1:**
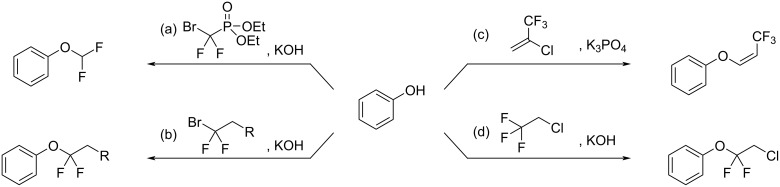
Methods for the synthesis of ethers containing fluorine substituents.

**Scheme 2:**
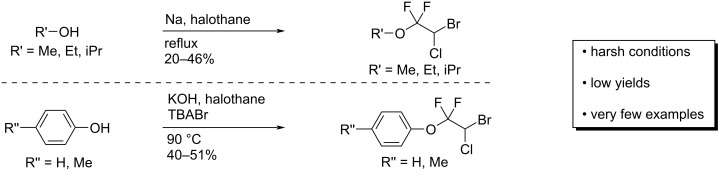
The previous work reported by Yagupol’skii et al.

Molecules containing fluoroalkyl groups are of interest in pharmaceutical and agrochemical sciences because deliberately incorporated fluorine atoms often change the chemical properties of the parent molecules by improving the absorption, resistance to metabolism, and pharmacological activities. To date, difluoromethyl or difluoromethylene compounds have been studied extensively as well as monofluorinated and trifluoromethylated arenes or aliphatics [1–4]. Recent progress in difluoromethylene chemistry successfully led to the finding of bioactive compounds such as pantoprazole, a proton pump inhibitor [5], and AFP-07, a prostaglandin I2 receptor-selective agonist [6–8], which suggests the importance of the difluoromethylene unit in drug discovery (Figure 1). There have been many reports for the construction of the difluoromethylene unit, such as the deoxygenating conversion of a carbonyl group to the difluoromethylene unit using *N*,*N*-diethylaminosulfur trifluoride (DAST) [9–14], the Reformatsky reaction of ethyl bromodifluoroacetate [15–23], and transformations of tetrafluoroethylene using suitable metal catalysts [24–31]. Additionally, recent advances in difluoromethylene chemistry have demonstrated the synthesis of aryl fluoroalkyl ethers as shown in Scheme 1 [32–34]. For example, the reactions of phenols with “*gem*-difluorocarbene precursors (route (a))” or “bromodifluoroalkyl compounds (route (b))” have been typically used to obtain a variety of aryl *gem*-difluoromethyl ethers. Particularly, the latter approach is useful for obtaining liquid crystal materials [35–38]. In the case of using 2-chloro-3,3,3-trifluoroprop-1-ene (route (c), Scheme 1), the aryl enol ether with a trifluoromethyl group was obtained [39–40]. A further example for the formation of an aryl fluoroalkyl ether was the reaction of phenol with 2-chloro-1,1,1-trifluoroethane, also known as HCFC-133a, in the presence of potassium hydroxide (KOH) to give the *gem*-difluoromethyl ethers along with the formation of 1-fluoro-2-chlorovinyl ether (route (d), Scheme 1) [41]. On the basis of our previous reports, we focused on halothane, 2-bromo-2-chloro-1,1,1-trifluoroethane, because the treatment of this compound with several bases was found to provide the highly electrophilic 2-bromo-2-chloro-1,1-difluoroethene [42–43]. Additionally, another report discussed the carbocationic character of the *gem*-difluorovinyl carbon that was explained by an orbital interaction between the n orbital (fluorine) and the π orbital [44]. A literature survey revealed that Yagupol’skii et al. have achieved the first synthesis of aryl or alkyl 2-bromo-2-chloro-1,1-difluoroethyl ethers by the reaction of alcohols with halothane as shown in Scheme 2 [45]. This study had a large impact on fluorine chemistry in terms of the availability of halothane to build difluoroalkyl ethers. However, these reactions have significant drawbacks such as the need of harsh reaction conditions, low chemical yields, and in particular, very few examples to understand the reaction profile (Scheme 2). To address this issue, we conducted extensive research to provide a general use of halothane for the construction of such difluoroalkyl ethers. In this paper, we discuss a new and practical synthetic approach to structurally unique and highly functionalized aryl 2-bromo-2-chloro-1,1-difluoroethyl ethers as well as several considerations of the reaction mechanism.

## Results and Discussion

We began our study to optimize the reaction conditions (Table 1). When two equivalents of halothane and sodium hydride (NaH) were treated with phenol (**1a**) at room temperature for 20 h, the reaction provided the ether product **2a** in 39% yield (Table 1, entry 1). In this case, some of the substrate phenol remained in the reaction mixture. Therefore, the reactions were conducted with an increased amount of halothane (2.5 and 4.0 equivalents). However, in both reactions, the yield of the product **2a** was not improved in spite of a significant decrease of the starting material, phenol (Table 1, entries 2 and 3). Next, as shown in entry 4 of Table 1, when the reaction was conducted with a lower loading of NaH, the desired product **2a** was obtained in the same yield as that in entry 1 (Table 1). From the result of entry 5 (Table 1) in which the reaction time was prolonged to 52 h, the reaction was considered to be sluggish because the yield of **2a** was only slightly better (55%). On the other hand, when the reaction temperature was raised to 40 or 60 °C, the product yield was gradually improved (Table 1, entries 6 and 7). It is worth noting that when changing the base to KOH, a dramatic improvement of the reaction efficiency was observed giving product **2a** in 74% yield, even though the reaction time was remarkably short (Table 1, entry 8). On the contrary, the reaction did not occur at all when potassium carbonate was used as a base, because the deprotonation of phenol became slow due to the low basicity (Table 1, entry 9).

**Table 1:** Optimization of the reaction conditions with phenol (**1a**).

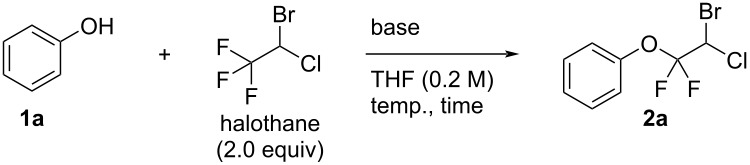				

entry	base (equiv)	temp. (°C)	time (h)	yield (%)^a^

1	NaH (2.0)	rt	20	39
2^b^	NaH (2.0)	rt	23	37
3^c^	NaH (2.0)	rt	23	32
4	NaH (1.5)	rt	24	39
5	NaH (1.5)	rt	52	55
6	NaH (1.5)	40	25	54
7	NaH (1.5)	60	13	74
8	KOH (1.5)	60	1.5	74
9	K_2_CO_3_ (1.5)	60	24	0

^a^Isolated yields. ^b^Halothane (2.5 equiv) was used. ^c^Halothane (4.0 equiv) was used.

With the optimal reaction conditions (Table 1, entry 8) in hand, we next examined the scope of the reaction (Table 2). When an electron-rich phenol, *p*-methoxyphenol (**1b**) was used, the reaction proceeded to afford ether **2b** in 79% yield (Table 2, entry 1). An o*rtho*-substitution with the bulkier *tert*-butyl group slightly affected the reaction giving product **2c** in still acceptable yield (47%, Table 2, entry 2). *p*-Nitrophenol (**1d**), which is an electron-poor phenol, was converted into the corresponding ether **2d** only in traces (Table 2, entry 3). By using 3.0 equivalents of KOH, *p*-trifluoromethylphenol (**1e**) provided the product **2e** in 48% yield (Table 2, entry 4). Also a phenol possessing a base-labile ester group (**1f**) afforded the product **2f** in 47% yield without the loss of the ester moiety (Table 2, entry 5). However, the reaction between *p*-hydroxybenzaldehyde (**1g**) and halothane did not proceed at all (Table 2, entry 6). A phenyl group in the *ortho* position (**1h**) did not affect the reaction and also the positional isomers **1i** and **1j**, gave the corresponding products in comparably high yields (Table 2, entries 7–9). Also, 1-naphthol (**1k**) was compatible with the reaction conditions giving the product **2k** in a good yield of 85% (Table 2, entry 10). As shown in entries 11–13 (Table 2), *o*-iodophenol (**1l**) and the alkenyl-substituted phenols **1m** and **1n**, all substrates that are susceptible to radical conditions, afforded the corresponding iodo- and alkenyl-substituted products, suggesting the reaction proceeds through an ionic process. 2-Hydroxychalcone (**1o**), which is a good Michael acceptor, also participated in the reaction to give the product **2o** in 56% yield, without byproduct formation from a Michael reaction (Table 2, entry 14). When *o*-aminophenol (**1p**) was used as the substrate, the coupling reaction occurred on the hydroxy group exclusively to give **2p** (Table 2, entry 15).

**Table 2:** The reaction scope with various phenols.

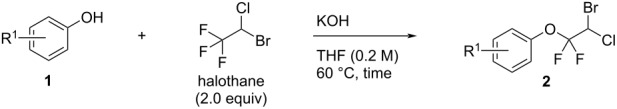				

entry	phenol	products	time (h)	yield (%)^a^

1	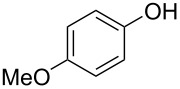 **1b**	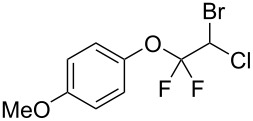 **2b**	2.5	79
2	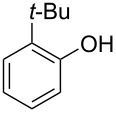 **1c**	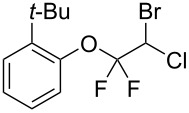 **2c**	0.5	47
3^b^	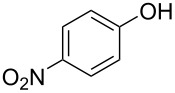 **1d**	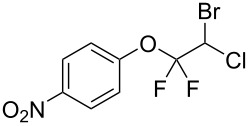 **2d**	20.5	3
4^b^	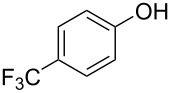 **1e**	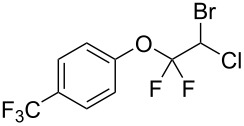 **2e**	7	48
5^b^	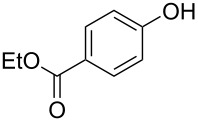 **1f**	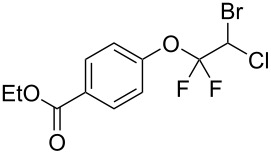 **2f**	13	47
6^b^	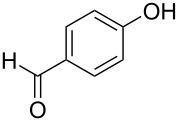 **1g**	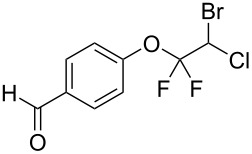 **2g**	24	no reaction
7	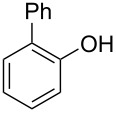 **1h**	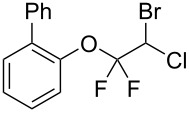 **2h**	3	71
8	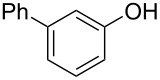 **1i**	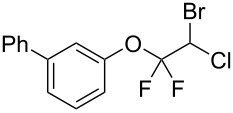 **2i**	1.5	79
9	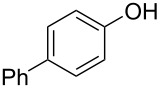 **1j**	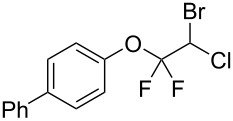 **2j**	5	88
10	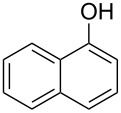 **1k**	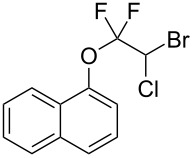 **2k**	3.5	85
11	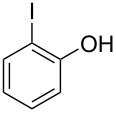 **1l**	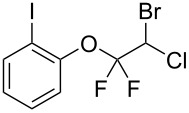 **2l**	6.5	67
12	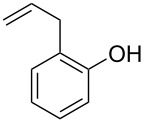 **1m**	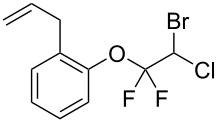 **2m**	1.5	81
13^c^	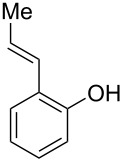 **1n**	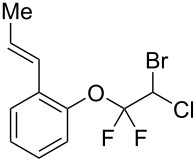 **2n**	1	71
14^b^	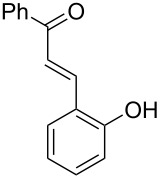 **1o**	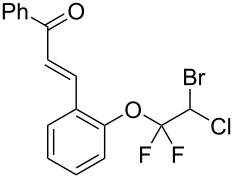 **2o**	20	56
15	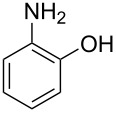 **1p**	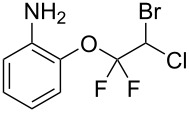 **2p**	19	51

^a^Isolated yield. ^b^KOH (3.0 equiv) was used. ^c^**1n** and **2n** were *cis*–*trans* mixtures.

**Scheme 3:**
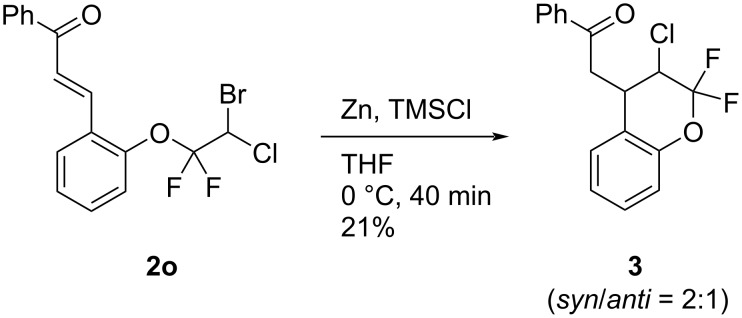
Intramolecular 1,4-addition of **2o**.

To show the synthetic advantages of the obtained *gem*-difluoro ethers **2**, we examined the further application of compound **2o** in a cyclization reaction. Upon the treatment of **2o** with zinc and chlorotrimethylsilane (TMSCl), the intramolecular 1,4-addition reaction of **2o** proceeded to give the 2,2-*gem*-difluorochromane **3** in low yield (Scheme 3).

**Scheme 4:**
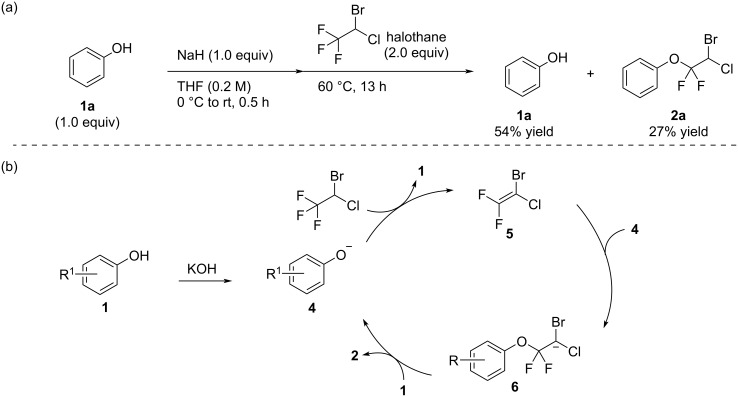
Proposed reaction mechanism.

Next, we moved on consideration of the reaction mechanism as shown in Scheme 4a. The reaction was carried out in a stepwise procedure in which the phenoxide was prepared by mixing equimolar amounts of phenol (**1a**) and NaH to consume all NaH in the reaction mixture, and then halothane was added to the reaction mixture. The stepwise reaction gave the product **2a** in 27% yield and **1a** was recovered in 54% (Scheme 4a). Based on these results, we speculated that the initially generated phenoxide **4** or potassium hydroxide remaining in the reaction mixture could deprotonate halothane to provide the fully halogenated ethylene **5** (Scheme 4b) [32–33,45]. Recent studies demonstrated an electrophilic character of a *gem*-difluorovinyl carbon due to the overlapping of the fluorine lone pairs and the adjacent π orbital in favor of the generation of a difluoromethyl cation species [44]. Considering these reports, the phenoxide attack on the *gem*-difluorovinyl carbon atom is a reasonable process to forward the reaction with the generation of carbanion **6**. Finally, the protonation of **6** by **1** or other acidic compounds, such as water molecules present in the reaction medium, would provide **2**.

## Conclusion

We exploited the reaction of various phenols with halothane to obtain aryl 2-bromo-2-chloro-1,1-difluoroethyl ethers. The products are structurally unique and represent highly functionalized compounds containing the *gem*-difluoroalkyl ether unit in addition to a bromochloromethyl group. The reaction proceeds under mild conditions and is compatible with variously substituted phenols giving the products in moderate to good yields. Additionally, the products are expected to undergo a variety of functionalization using ionic and radical processes. Further studies on the chemical transformations of the ether products are currently underway.

## Experimental

### General information

^1^H NMR, ^13^C NMR and ^19^F NMR spectra were recorded on JEOL ECZ 400S spectrometers. Chemical shifts of ^1^H NMR are reported in ppm from tetramethylsilane (TMS) as an internal standard. Chemical shifts of ^13^C NMR are reported in ppm from the center line of the triplet at 77.16 ppm for deuteriochloroform. Chemical shifts of ^19^F NMR are reported in ppm from CFCl_3_ as an internal standard. All data are reported as follows: chemical shifts, multiplicity (s = singlet, d = doublet, t = triplet, q = quartet, sep = septet, br = broad, brd = broad-doublet, m = multiplet), coupling constants (Hz), relative integration value. Mass spectra were obtained on a JEOL JMS-700T spectrometer (EI).

### Materials

All commercially available materials were used as received without further purification. All experiments were carried out under argon atmosphere in flame-dried glassware using standard inert techniques for introducing reagents and solvents unless otherwise noted.

### Typical procedure for the reaction between various phenols and halothane

To a solution of the phenol (1.0 mmol) in THF (5.0 mL) was added previously ground KOH (1.5 mmol) and halothane (2.0 mmol) in small portions at 0 °C. The solution was heated to 60 °C until the reaction was completed. Then, the reaction mixture was quenched by the addition of sat. aq. NH_4_Cl (20 mL) at 0 °C and extracted with AcOEt. The organic phase was washed with brine (30 mL), dried over Na_2_SO_4_, filtered, and concentrated under reduced pressure. The residue was purified by column chromatography to afford the products **2**.

**2-Bromo-2-chloro-1,1-difluoroethyl phenyl ether (2a):** The title product **2a** was purified by column chromatography and preparative TLC (hexane only) and obtained in 74% yield (199.6 mg). Pale yellow oil; ^1^H NMR (400 MHz, CDCl_3_) δ 5.91 (t, *J* = 5.2 Hz, 1H), 7.20–7.31 (m, 5H), 7.35–7.42 (m, 2H); ^13^C NMR (100 MHz, CDCl_3_) δ 53.5 (t, *J* = 41.7 Hz), 119.7 (t, *J* = 267.1 Hz), 121.8, 126.5, 129.7, 149.7; ^19^F NMR (376 MHz, CDCl_3_) δ −77.9 (dd, *J* = 136.9, 5.2 Hz, 1F), −78.2 (dd, *J* = 136.9, 5.2 Hz, 1F); EIMS (*m/z*): 270, 272 [M]^+^; HRMS–EI (*m*/*z*): [M]^+^ calcd for C_8_H_6_BrClF_2_O, 269.9259, 271.9238; found, 269.9264, 271.9233.

**4-Benzoylmethyl-3-chloro-2,2-difluorochromane (3):** To a solution of **2o** (210.8 mg, 0.53 mmol) in THF (2.6 mL) was added zinc (42.1 mg, 0.64 mmol) and chlorotrimethylsilane (133 μL, 1.05 mmol) at 0 °C. After stirring for 40 min, the reaction mixture was quenched by the addition of 10% aq. HCl (40 mL) at 0 °C and extracted with AcOEt. The organic phase was dried over Na_2_SO_4_, filtered, and concentrated under reduced pressure. The residue was purified by column chromatography (hexane/CHCl_3_ 4:1 to 1:1) and product **3** was obtained as a mixture of two diastereoisomers (*syn*/*anti* 2:1) in 21% yield (36.9 mg). Yellow oil; ^1^H NMR (400 MHz, CDCl_3_) δ 3.48 (ddd, *J* = 18.7, 5.2, 1.4 Hz, *anti*-isomer), 3.73 (ddd, *J* = 18.7, 7.0, 1.2 Hz, *anti*-isomer, 2H), 3.57–3.71 (m, *syn*-isomer, 2H), 4.04–4.18 (m, *anti*-isomer, 1H), 4.29–4.37 (m, *syn*-isomer, 1H), 4.66 (ddd, *J* = 7.0, 6.0, 1.0 Hz, *anti*-isomer, 1H), 4.82 (t, *J* = 4.1 Hz, *syn*-isomer, 1H), 7.01–7.22 (m, 3H), 7.24–7.33 (m, 1H), 7.49 (t, *J* = 7.3 Hz, *anti*-isomer, 2H), 7.52 (t, *J* = 7.3 Hz, *syn*-isomer, 2H), 7.58–7.67 (m, 1H), 7.97 (dd, *J* = 7.8, 1.3 Hz, *anti*-isomer, 2H), 8.05 (dd, *J* = 7.8, 1.3 Hz, *syn*-isomer, 2H); ^13^C NMR (100 MHz, CDCl_3_) δ 34.5 (*syn*-isomer), 38.4 (d, *J* = 2.2 Hz, *syn*-isomer), 39.3 (*anti*-isomer), 41.7 (d, *J* = 6.0 Hz, *anti*-isomer), 54.7 (d, *J* = 28.6 Hz, *anti*-isomer), 54.9 (d, *J* = 26.6 Hz, *syn*-isomer), 55.1 (d, *J* = 28.6 Hz, *anti*-isomer), 55.3 (d, *J* = 26.6 Hz, *syn*-isomer), 117.3 (*syn*-isomer), 117.4 (*anti*-isomer), 119.49 (d, *J* = 256.9 Hz, *anti*-isomer), 119.52 (d, *J* = 251.1 Hz, *syn*-isomer), 120.6 (*syn*-isomer), 121.5 (*anti*-isomer), 122.06 (d, *J* = 256.9 Hz, *anti*-isomer), 122.11 (d, *J* = 251.1 Hz, *syn*-isomer), 124.1 (*syn*-isomer), 124.3 (*anti*-isomer), 127.0 (*anti*-isomer), 128.2 (*syn*-isomer), 128.3, 129.0, 129.1 (*anti*-isomer), 129.3 (*syn*-isomer), 133.9, 136.4 (*anti*-isomer), 136.5 (*syn*-isomer), 149.2 (*anti*-isomer), 149.6 (d, *J* = 4.1 Hz, *syn*-isomer), 197.0 (*anti*-isomer), 197.3 (*syn*-isomer); ^19^F NMR (376 MHz, CDCl_3_) δ −69.0 (dd, *J* = 155.8, 4.1 Hz, *syn*-isomer, 1F), −70.8 (d, *J* = 157.3 Hz, *anti*-isomer, 1F), −77.6 (ddd, *J* = 157.6, 7.0, 4.2 Hz, *anti*-isomer, 1F), −79.0 (dd, *J* = 155.8, 3.1 Hz, *syn*-isomer, 1F); EIMS (*m*/*z*): 322 [M]^+^; HRMS–EI (*m*/*z*): [M]^+^ calcd for C_17_H_13_ClF_2_O_2_, 322.0572; found, 322.0565.

## Supporting Information

File 1 Characterization data for **2b**–**p** and copies of ^1^H, ^13^C, and ^19^F NMR spectra.
