# Supplementary material for: Synthesis of aryl 2-bromo-2-chloro-1,1-difluoroethyl ethers through the base-mediated reaction between phenols and halothane
Source: Beilstein J Org Chem. 2021 Jan 11;17:89–96. doi: 10.3762/bjoc.17.9 (PMC7814179; doi:10.3762/bjoc.17.9)
Supplement: File 1 — Characterization data for 2b–p and copies of 1H, 13C, and 19F NMR spectra. [file Beilstein_J_Org_Chem-17-89-s001.pdf]

BEILSTEIN JOURNAL OF ORGANIC CHEMISTRY

Supporting Information

for

Synthesis of aryl 2-bromo-2-chloro-1,1-difluoroethyl ethers through the base-mediated reaction between phenols and halothane

Yukiko Karuo, Ayaka Kametani, Atsushi Tarui, Kazuyuki Sato, Kentaro Kawai and Masaaki Omote

Beilstein J. Org. Chem. **2021,** *17,* 89–96. doi:10.3762/bjoc.17.9

Characterization data for 2b–p and copies of ^{1}H, ^{13}C, and ^{19}F NMR spectra

License and Terms: This is a supporting information file under the terms of the Creative Commons Attribution License (https://creativecommons.org/licenses/by/4.0). Please note that the reuse, redistribution and reproduction in particular requires that the author(s) and source are credited and that individual graphics may be subject to special legal provisions.
The license is subject to the *Beilstein Journal of Organic Chemistry* terms and conditions: (https://www.beilstein-journals.org/bjoc/terms)

Table of contents

1. Characterization data for compounds **2b**–**p** S2–S4

2. ^{1}H, ^{13}C and ^{19}F NMR spectra S5–S28

1. Characterization data for 2b–p

2-Bromo-2-chloro-1,1-difluoroethyl *p*-methoxyphenyl ether (2b): The title product (**2b**) was purified by column chromatography (hexane:$CHCl_3$ = 19:1 to hexane:AcOEt = 9:1) and was obtained in 79% yield (237.2 mg). A yellow oil; ^{1}H NMR (400 MHz, $CDCl_3$) δ: 3.81 (3H, s), 5.90 (1H, t, *J* = 5.0 Hz), 6.88 (2H, d, *J* = 9.1 Hz), 7.15 (2H, d, *J* = 9.1 Hz); ^{13}C NMR (100 MHz, $CDCl_3$) δ: 53.5 (t, *J* = 42.1 Hz), 55.7, 114.6, 119.7 (t, *J* = 267.1 Hz), 123.2, 142.9, 157.9; ^{19}F NMR (376 MHz, $CDCl_3$) δ: -78.2 (1F, dd, *J* = 137.5, 5.0 Hz), -78.5 (1F, dd, *J* = 137.5, 5.0 Hz); MS (EI) *m/z*: 300 (M^+); HRMS (EI) Calcd. for $C_9H_8BrClF_2O_2$: 299.9364 (M^+), Found: 299.9361.

2-Bromo-2-chloro-1,1-difluoroethyl *o-t*-butylphenyl ether (2c): The title product (**2c**) was purified by column chromatography and preparative TLC (hexane only). **2c** was obtained in 47% yield (155.2 mg). A colorless oil; ^{1}H NMR (400 MHz, $CDCl_3$) δ: 1.41 (9H, s), 5.97 (1H, t, *J* = 5.2 Hz), 7.13 (1H, ddd, *J* = 7.5, 7.5, 1.2 Hz), 7.18-7.24 (1H, m), 7.37-7.43 (2H, m); ^{13}C NMR (100 MHz, $CDCl_3$) δ: 30.5, 35.0, 54.0 (t, *J* = 42.1 Hz), 119.1 (t, *J* = 3.6 Hz), 120.0 (t, *J* = 270.8 Hz), 125.0, 127.2, 127.8, 140.0, 149.6; ^{19}F NMR (376 MHz, $CDCl_3$) δ: -76.9 (1F, ddd, *J* = 134.3, 5.2, 2.1 Hz), -77.2 (1F, ddd, *J* = 134.3, 5.2, 2.1 Hz); MS (EI) *m/z*: 326 (M^+); HRMS (EI) Calcd. for $C_{12}H_{14}BrClF_2O$: 325.9885 (M^+), Found: 325.9879.

2-Bromo-2-chloro-1,1-difluoroethyl *p*-nitrophenyl ether (2d): The title product (**2d**) was purified by column chromatography and preparative TLC (hexane:AcOEt = 19:1). **2d** was obtained in 3% yield (10.7 mg). A yellow oil; ^{1}H NMR (400 MHz, $CDCl_3$) δ: 5.95 (1H, t, *J* = 5.1 Hz), 7.39 (2H, d, *J* = 8.4 Hz), 8.29 (2H, d, *J* = 8.4 Hz); ^{13}C NMR (100 MHz, $CDCl_3$) δ: 52.9 (t, *J* = 40.6 Hz), 119.8 (t, *J* = 272.0 Hz), 121.8, 125.7, 145.8, 154.4; ^{19}F NMR (376 MHz, $CDCl_3$) δ: -78.1 (1F, dd, *J* = 134.9, 4.9 Hz), -78.5 (1F, dd, *J* = 134.9, 4.9 Hz); MS (EI) *m/z*: 315 (M^+); HRMS (EI) Calcd. for $C_8H_5BrClF_2NO_3$: 314.9109 (M^+), Found: 314.9106.

2-Bromo-2-chloro-1,1-difluoroethyl *p*-trifluoromethylphenyl ether (2e): The title product (**2e**) was purified by column chromatography (hexane only). **2e** was obtained in 48% yield (163.3 mg). A pale yellow oil; ^{1}H NMR (400 MHz, $CDCl_3$) δ: 5.93 (1H, t, *J* = 5.0 Hz), 7.35 (2H, d, *J* = 8.3 Hz), 7.67 (2H, d, *J* = 8.3 Hz); ^{13}C NMR (100 MHz, $CDCl_3$) δ: 53.1 (t, *J* = 40.8 Hz), 119.8 (t, *J* = 270.2 Hz), 121.9, 123.9 (q, *J* = 272.3 Hz), 127.2 (q, *J* = 3.5 Hz), 128.8 (q, *J* = 33.0 Hz), 152.2; ^{19}F NMR (376 MHz, $CDCl_3$) δ: -62.2 (3F, s), -78.0 (1F, dd, *J* = 136.2, 4.9 Hz), -78.3 (1F, dd, *J* = 136.2, 4.9 Hz); MS (EI) *m/z*: 338 (M^+); HRMS (EI) Calcd. for $C_9H_5BrClF_5O$: 337.9132 (M^+), Found: 337.9135.

Ethyl *p*-(2-bromo-2-chloro-1,1-difluoroethoxy)benzoate (2f): The title product (**2f**) was purified by column chromatography and preparative TLC (hexane:AcOEt = 15:1). **2f** was obtained in 47% yield (161.0 mg). A pale yellow oil; ^{1}H NMR (400 MHz, $CDCl_3$) δ: 1.40 (3H, t, *J* = 7.1 Hz), 4.39 (2H, q, *J* = 7.1 Hz), 5.92 (3H, t, *J* = 5.1 Hz), 7.29 (2H, d, *J* = 8.4 Hz), 8.08 (2H, d, *J* = 8.4 Hz); ^{13}C NMR (100 MHz, $CDCl_3$) δ: 14.5, 53.3 (t, *J* = 41.2 Hz), 61.3, 119.7 (t, *J* = 270.1 Hz), 121.2 , 128.7, 131.5, 153.2, 165.7; ^{19}F NMR (376 MHz, $CDCl_3$) δ: -78.0 (1F, dd, *J* = 136.5, 5.1 Hz), -78.3 (1F, dd, *J* = 136.5, 5.1 Hz); MS (EI) *m/z*: 342 (M^+); HRMS (EI) Calcd. for $C_{11}H_{10}BrClF_2O_3$: 341.9470 (M^+), Found: 341.9473.

2-Bromo-2-chloro-1,1-difluoroethyl *o*-phenylphenyl ether (2h): The title product (**2h**) was purified by column chromatography and preparative TLC (hexane only). **2h** was obtained in 71% yield (247.1 mg). A colorless oil; ^{1}H NMR (400 MHz, $CDCl_3$) δ: 5.64 (1H, t, *J* = 5.2 Hz), 7.30-7.49 (9H, m); ^{13}C NMR (100 MHz, $CDCl_3$) δ: 53.4 (t, *J* = 41.2 Hz), 119.6 (t, *J* = 268.8 Hz), 122.4, 126.7, 127.6, 128.2, 128.6, 129.7, 131.5, 135.9, 137.4, 146.7; ^{19}F NMR (376

MHz, $CDCl_3$) δ: -77.5 (2F, dd, *J* = 5.2, 3.1 Hz); MS (EI) *m/z*: 346 (M^+); HRMS (EI) Calcd. for $C_{14}H_{10}BrClF_2O$: 345.9572 (M^+), Found: 345.9562.

2-Bromo-2-chloro-1,1-difluoroethyl *m*-phenylphenyl ether (2i): The title product (**2i**) was purified by column chromatography and preparative TLC (hexane only). **2i** was obtained in 79% yield (275.9 mg). A pale yellow oil; ^{1}H NMR (400 MHz, $CDCl_3$) δ: 5.94 (1H, t, *J* = 5.1 Hz), 7.19-7.24 (1H, m), 7.35-7.53 (6H, m), 7.56-7.61 (2H, m); ^{13}C NMR (100 MHz, $CDCl_3$) δ: 53.5 (t, *J* = 41.7 Hz), 119.7 (t, *J* = 269.6 Hz), 120.5, 120.6, 125.3, 127.3, 128.0, 129.1, 130.0, 140.0, 143.2, 150.0; ^{19}F NMR (376 MHz, $CDCl_3$) δ: -77.7 (1F, dd, *J* = 136.9, 5.1 Hz), -78.1 (1F, dd, *J* = 136.9, 5.1 Hz); MS (EI) *m/z*: 346 (M^+); HRMS (EI) Calcd. for $C_{14}H_{10}BrClF_2O$: 345.9572 (M^+), Found: 345.9567.

2-Bromo-2-chloro-1,1-difluoroethyl *p*-phenylphenyl ether (2j): The title product (**2j**) was purified by column chromatography (hexane only) and was obtained in 88% yield (304.4 mg). White solid; mp: 46.0-46.5 °C; ^{1}H NMR (400 MHz, $CDCl_3$) δ: 5.93 (1H, t, *J* = 4.8 Hz), 7.30 (2H, d, *J* = 7.9 Hz), 7.37 (1H, t, *J* = 7.2 Hz), 7.45 (2H, dd, *J* = 7.9, 7.2 Hz), 7.58 (4H, t, *J* = 8.4 Hz); ^{13}C NMR (100 MHz, $CDCl_3$) δ: 53.5 (t, *J* = 41.6 Hz), 119.7 (t, *J* = 267.7 Hz), 122.1, 127.2, 127.7, 128.4, 129.0, 139.7, 140.1, 149.1; ^{19}F NMR (376 MHz, $CDCl_3$) δ: -78.0 (1F, dd, *J* = 137.1, 4.8 Hz), -78.1 (1F, dd, *J* = 137.1, 4.8 Hz); MS (EI) *m/z*: 346 (M^+); HRMS (EI) Calcd. for $C_{14}H_{10}BrClF_2O$: 345.9572 (M^+), Found: 345.9566.

2-Bromo-2-chloro-1,1-difluoroethyl 1-naphthyl ether (2k): The title product (**2k**) was purified by column chromatography (hexane only). **2k** was obtained in 85% yield (275.3 mg). A colorless oil.; ^{1}H NMR (400 MHz, $CDCl_3$) δ: 6.09 (1H, t, *J* = 4.8 Hz), 7.39-7.49 (2H, m), 7.51-7.60 (2H, m), 7.74-7.80 (1H, m), 7.84-7.91 (1H, m), 8.21-8.29 (1H, m); ^{13}C NMR (100 MHz, $CDCl_3$) δ: 53.7 (t, *J* = 41.7 Hz), 117.4, 120.1 (t, *J* = 268.1 Hz), 122.0, 125.3, 126.5, 126.8, 126.9, 127.7, 127.9, 134.8, 145.5; ^{19}F NMR (376 MHz, $CDCl_3$) δ: -77.5 (1F, dd, *J* = 136.6, 4.8 Hz), -78.0 (1F, dd, *J* = 136.6, 4.8 Hz); MS (EI) *m/z*: 320 (M^+); HRMS (EI) Calcd. for $C_{12}H_8BrClF_2O$: 319.9415 (M^+), Found: 319.9416.

2-Bromo-2-chloro-1,1-difluoroethyl *o*-iodophenyl ether (2l): The title product (**2l**) was purified by column chromatography (hexane only) and was obtained in 67% yield (266.7 mg). A yellow oil; ^{1}H NMR (400 MHz, $CDCl_3$) δ: 6.01 (1H, t, *J* = 5.6 Hz), 6.97-7.03 (1H, m), 7.30-7.40 (2H, m), 7.86 (1H, dd, *J* = 8.4, 1.6 Hz); ^{13}C NMR (100 MHz, $CDCl_3$) δ: 53.5 (t, *J* = 40.3 Hz), 89.9, 119.9 (t, *J* = 270.5 Hz), 122.0, 127.9, 129.6, 140.3, 149.9; ^{19}F NMR (376 MHz, $CDCl_3$) δ: -77.4 (1F, ddd, *J* = 135.0, 5.8, 1.1 Hz), -77.8 (1F, ddd, *J* = 135.0, 5.8, 1.1 Hz); MS (EI) *m/z*: 396 (M^+); HRMS (EI) Calcd. for $C_8H_5BrClF_2IO$: 395.8225 (M^+), Found: 395.8231.

***o*-Allylphenyl 2-bromo-2-chloro-1,1-difluoroethyl ether (2m):** The title product (**2m**) was purified by column chromatography (hexane only) and was obtained in 81% yield (253.4 mg). A colorless oil; ^{1}H NMR (400 MHz, $CDCl_3$) δ: 3.49 (2H, d, *J* = 6.8 Hz), 5.06-5.14 (2H, m), 5.95 (1H, t, *J* = 4.6 Hz), 5.96 (1H, ddt, *J* = 17.6, 9.4, 6.7 Hz), 7.17-7.32 (4H, m); ^{13}C NMR (100 MHz, $CDCl_3$) δ: 34.2, 53.7 (t, *J* = 41.8 Hz), 116.5 , 119.9 (t, *J* = 268.9 Hz), 121.8, 126.5, 127.5, 130.8, 133.2, 136.2, 147.7; ^{19}F NMR (376 MHz, $CDCl_3$) δ: -77.3 (1F, dd, *J* = 137.1, 4.6 Hz), -77.6 (1F, dd, *J* = 137.1, 4.6 Hz); MS (EI) *m/z*: 310 (M^+); HRMS (EI) Calcd. for $C_{11}H_{10}BrClF_2O$: 309.9572 (M^+), Found: 309.9568.

2-Bromo-2-chloro-1,1-difluoroethyl *o*-propenylphenyl ether (2n): The title product (**2n**) was purified by column chromatography and preparative TLC (hexane only). **2n** was obtained an inseparable mixture of *cis*-*trans* isomers (1:2.2) in 71% yield (219.9 mg). A colorless oil; ^{1}H NMR (400 MHz, $CDCl_3$) δ: 1.81 (dd, *J* = 7.0, 1.7 Hz, *cis*-isomer)

and 1.91 (dd, *J* = 6.7, 1.7 Hz, *trans*-isomer) (3H), 5.88 (dq, *J* = 11.4, 7.0 Hz, *cis*-isomer) and 6.26 (dd, *J* = 15.7, 6.7 Hz, *trans*-isomer) (1H), 5.93 (t, *J* = 5.0 Hz, *cis*-isomer) and 5.98 (t, *J* = 4.8 Hz, *trans*-isomer) (1H), 6.54 (dq, *J* = 11.4, 1.7 Hz, *cis*-isomer) and 6.76 (d, *J* = 15.7 Hz, *trans*-isomer) (1H), 7.17-7.31 (3H, m) , 7.33-7.38 (m, *cis*-isomer) and 7.49-7.57 (m, *trans*-isomer) (1H); ^{13}C NMR (100 MHz, $CDCl_3$) δ: 14.7 (*cis*-isomer), 19.0 (*trans*-isomer), 53.6 (t, *J* = 41.8 Hz, *cis*-isomer), 53.7 (t, *J* = 41.8 Hz, *trans*-isomer), 119.77 (t, *J* = 267.9 Hz, *cis*-isomer), 119.81 (t, *J* = 268.0 Hz, *trans*-isomer), 122.2 (*cis*-isomer), 122.4 (*trans*-isomer), 124.5, 126.0 (*cis*-isomer), 126.4 (*trans*-isomer), 126.6 (*trans*-isomer), 127.7 (*trans*-isomer), 127.9 (*cis*-isomer), 128.4 (*trans*-isomer), 128.8 (*cis*-isomer), 130.9 (*cis*-isomer), 131.4 (*cis*-isomer), 131.9 (*trans*-isomer), 146.1 (*trans*-isomer), 147.2 (*cis*-isomer); ^{19}F NMR (376 MHz, $CDCl_3$) δ: -77.2 (1F, dd, *J* = 137.1, 4.8 Hz, *trans*-isomer), -77.6 (1F, dd, *J* = 137.1, 4.8 Hz, *trans*-isomer), -77.51 (1F, dd, *J* = 9.0, 5.0 Hz, *cis*-isomer), -77.53 (1F, dd, *J* = 9.0, 5.0 Hz, *cis*-isomer); MS (EI) *m*/*z*: 310 (M^+); HRMS (EI) Calcd. for $C_{11}H_{10}BrClF_2O$: 309.9572 (M^+), Found: 309.9568.

(*E*)-2'-(2-Bromo-2-chloro-1,1-difluoroethoxy)-chalcone (2o): The title product (**2o**) was purified by column chromatography and preparative TLC (hexane: AcOEt = 9:1). **2o** was obtained in 56% yield (225.0 mg). A yellow solid; mp: 66.5-67.1 °C; ^{1}H NMR (400 MHz, $CDCl_3$) δ: 5.97 (1H, t, *J* = 4.8 Hz), 7.32-7.39 (2H, m), 7.41-7.54 (4H, m), 7.55-7.62 (1H, m), 7.79-7.84 (1H, m), 7.96-8.01 (2H, m), 8.14 (1H, d, *J* = 16.0 Hz); ^{13}C NMR (100 MHz, $CDCl_3$) δ: 53.2 (t, *J* = 41.0 Hz), 119.9 (t, *J* = 269.4 Hz), 122.7, 125.0, 126.9, 127.7, 128.6, 128.8, 129.0, 131.5, 132.9, 138.1, 138.5, 148.4, 191.2; ^{19}F NMR (376 MHz, $CDCl_3$) δ: -77.2 (1F, dd, *J* = 136.4, 4.8 Hz), -77.8 (1F, dd, *J* = 136.4, 4.8 Hz); MS (EI) *m*/*z*: 400 (M^+); HRMS (EI) Calcd. for $C_{17}H_{12}BrClF_2O_2$: 399.9677 (M^+), Found: 399.9670.

***o*-Aminophenyl 2-bromo-2-chloro-1,1-difluoroethyl ether (2p):** The title product (**2p**) was purified by column chromatography (hexane: AcOEt = 9:1) and was obtained in 51% yield (145.3 mg). A yellow oil; ^{1}H NMR (400 MHz, $CDCl_3$) δ: 3.93 (2H, br s), 5.98 (1H, t, *J* = 4.4 Hz), 6.72 (1H, dd, *J* = 7.6, 1.2 Hz), 6.79 (1H, dd, *J* = 8.0, 1.6 Hz), 7.06 (1H, dd, *J* = 7.6, 1.6 Hz), 7.16 (2H, dd, *J* = 8.0, 1.2 Hz); ^{13}C NMR (100 MHz, $CDCl_3$) δ: 53.7 (t, *J* = 41.9 Hz), 116.7, 118.4, 120.2 (t, *J* = 267.4 Hz), 122.6, 127.4, 136.5, 139.9; ^{19}F NMR (376 MHz, $CDCl_3$) δ: -77.7 (1F, dd, *J* = 137.1, 4.4 Hz), -78.5 (1F, dd, *J* = 137.1, 4.4 Hz); MS (EI) *m*/*z*: 285 (M^+); HRMS (EI) Calcd. for $C_8H_7BrClF_2NO$: 284.9368 (M^+), Found: 284.9363.

2. ^{1}H, ^{13}C and ^{19}F NMR spectra

2-Bromo-2-chloro-1,1-difluoroethyl phenyl ether (2a)

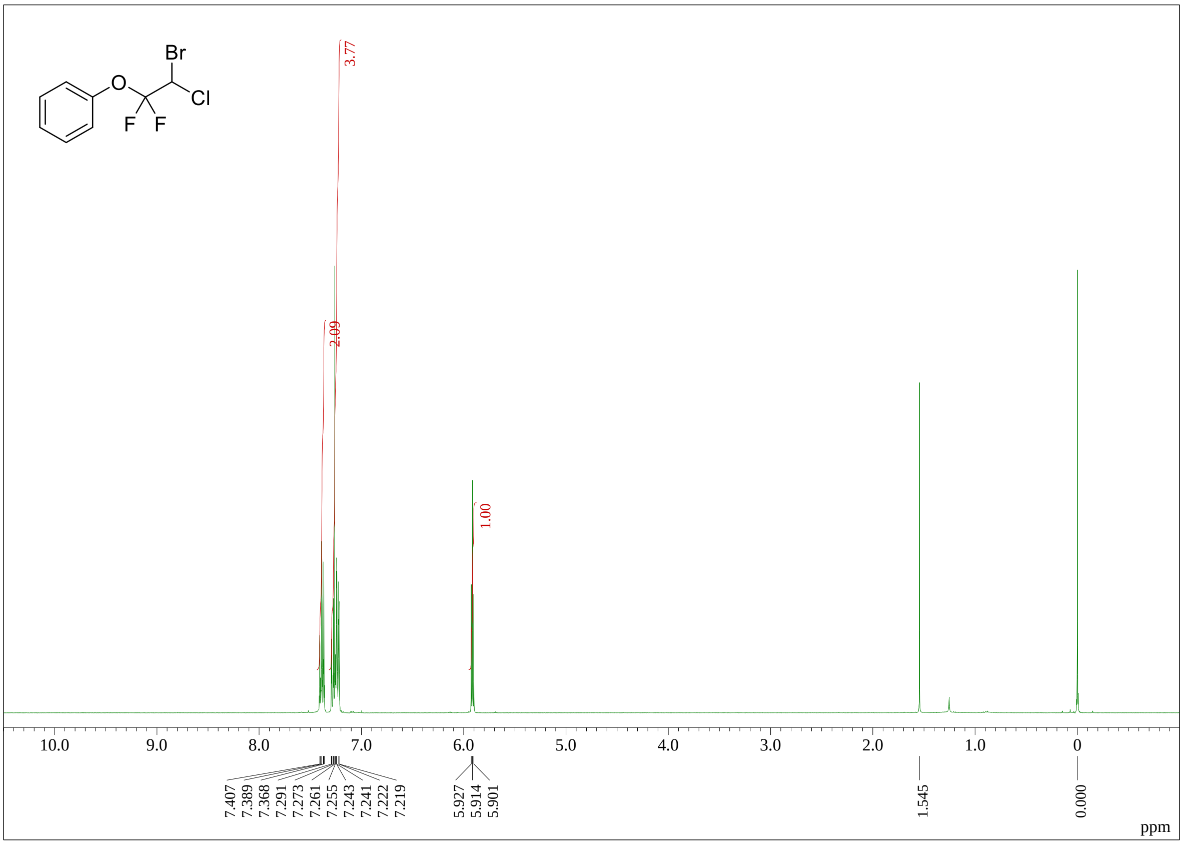

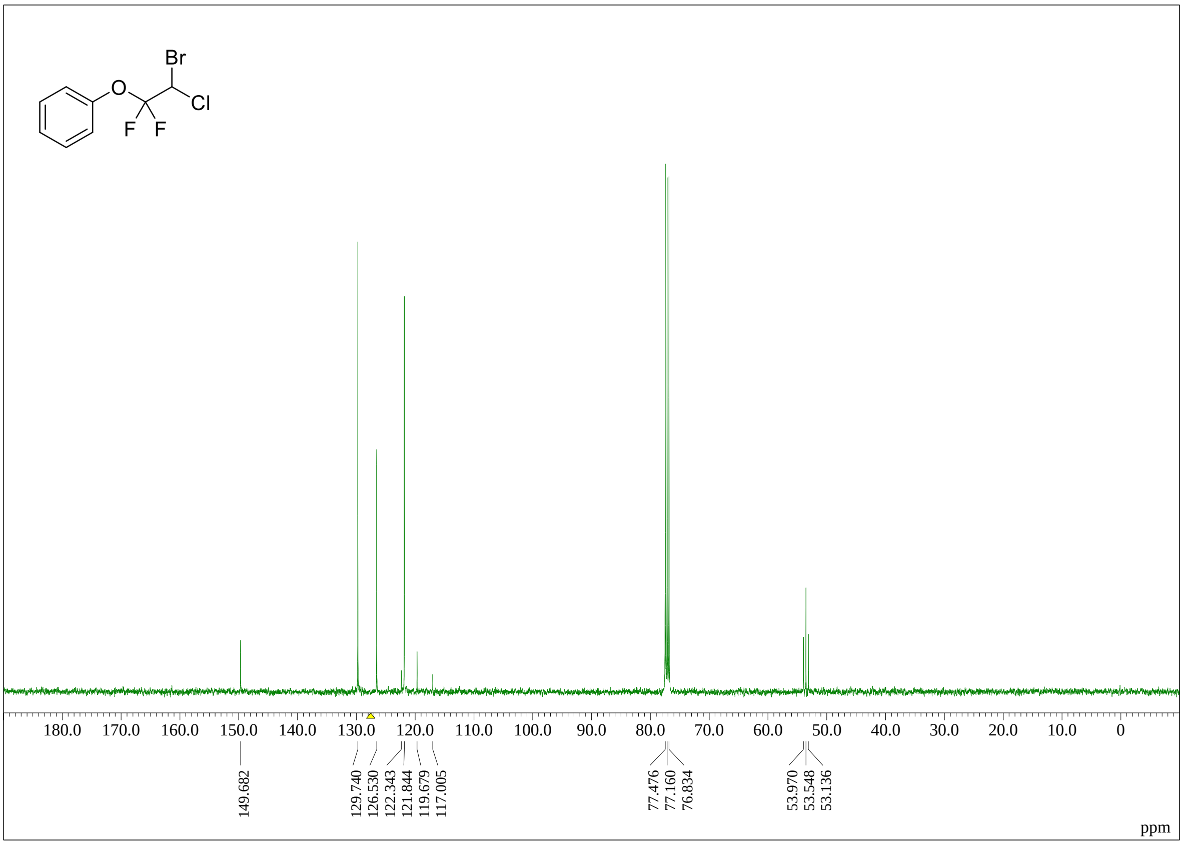

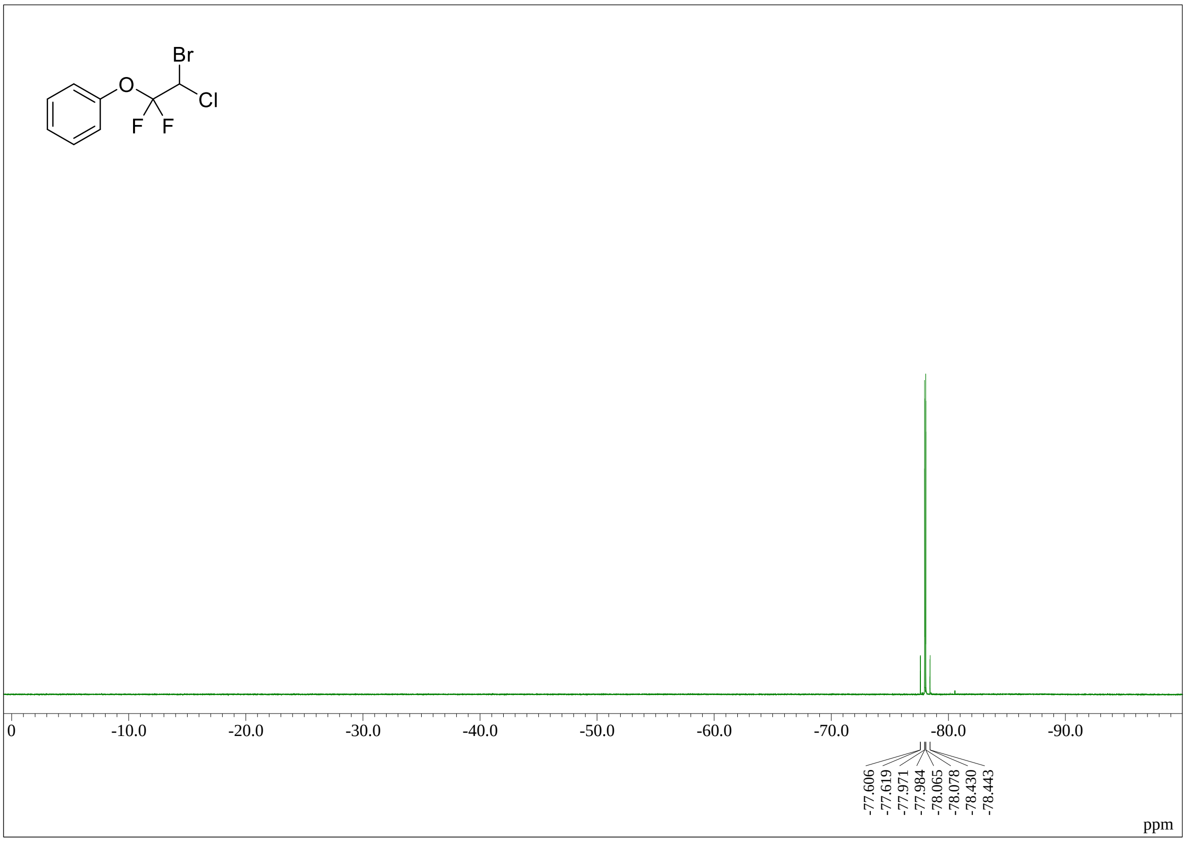

2-Bromo-2-chloro-1,1-difluoroethyl *p*-methoxyphenyl ether (2b)

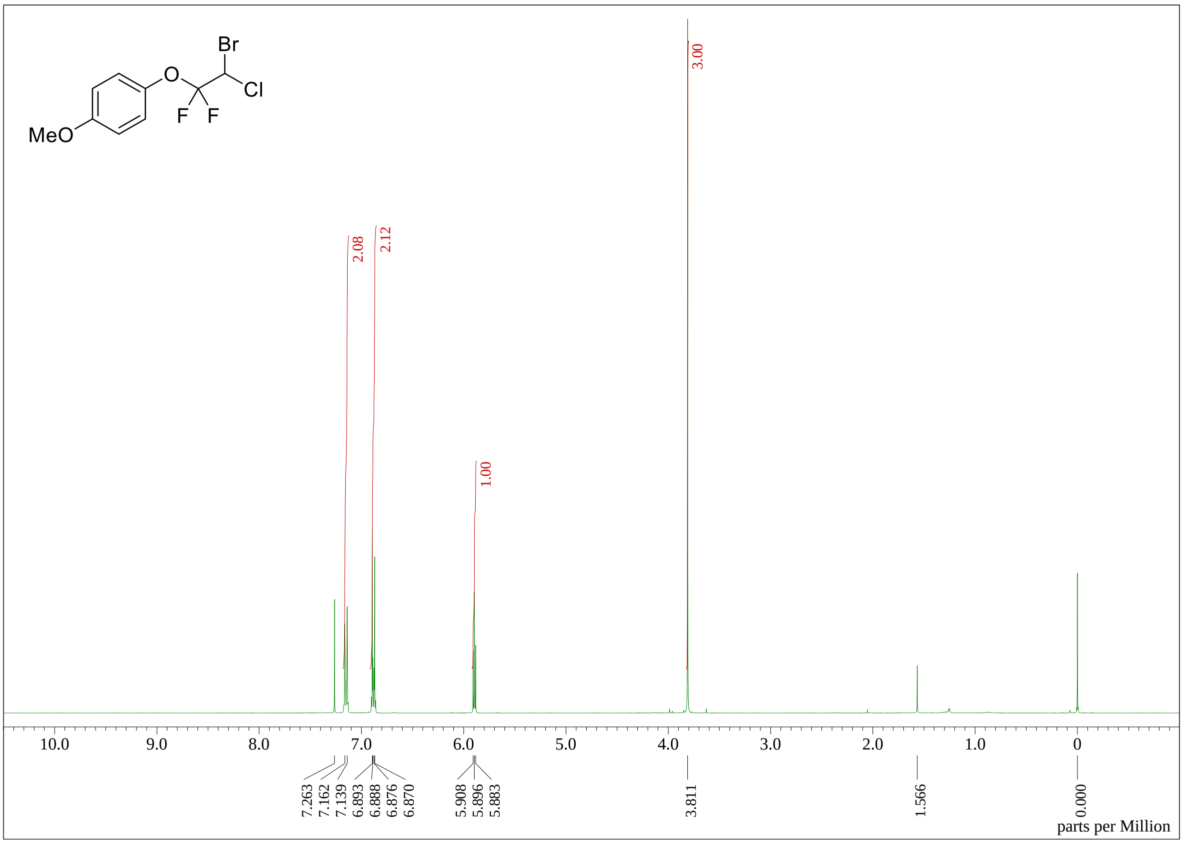

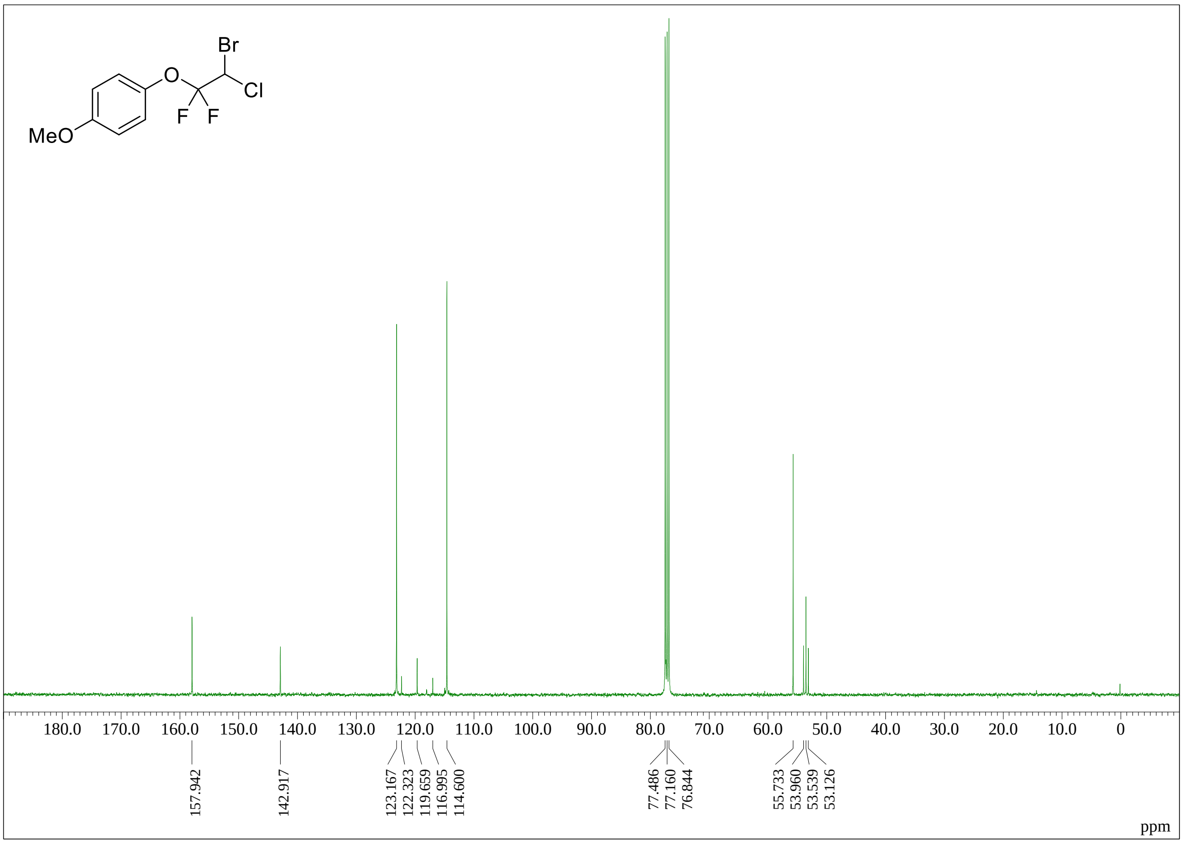
Br
O
Cl
F F
MeO
180.0 170.0 160.0 150.0 140.0 130.0 120.0 110.0 100.0 90.0 80.0 70.0 60.0 50.0 40.0 30.0 20.0 10.0 0
157.942
142.917
123.167
122.323
119.659
116.995
114.600
77.486
77.160
76.844
55.733
53.960
53.539
53.126
ppm

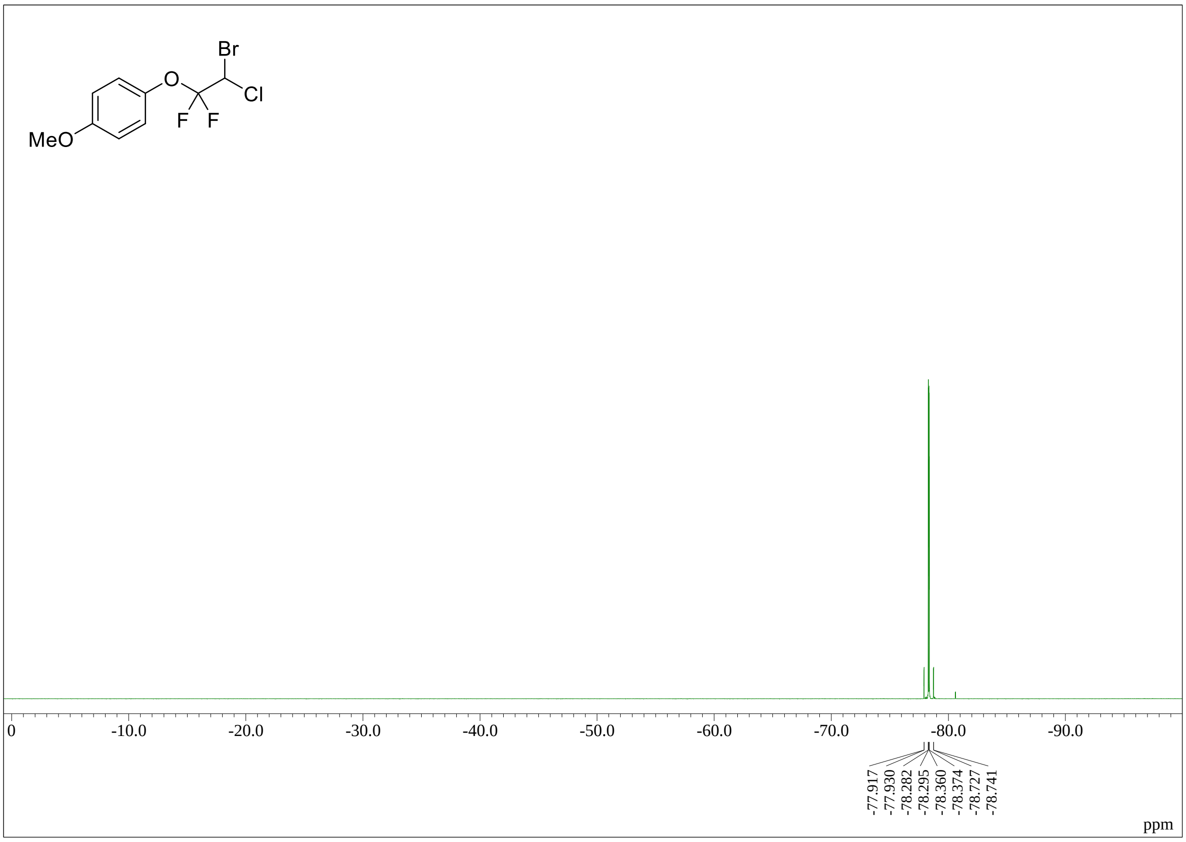
Br
O
Cl
F F
MeO
0 -10.0 -20.0 -30.0 -40.0 -50.0 -60.0 -70.0 -80.0 -90.0
-77.917
-77.930
-78.282
-78.295
-78.360
-78.374
-78.727
-78.741
ppm

2-Bromo-2-chloro-1,1-difluoroethyl *o*-*t*-butylphenyl ether (2c)

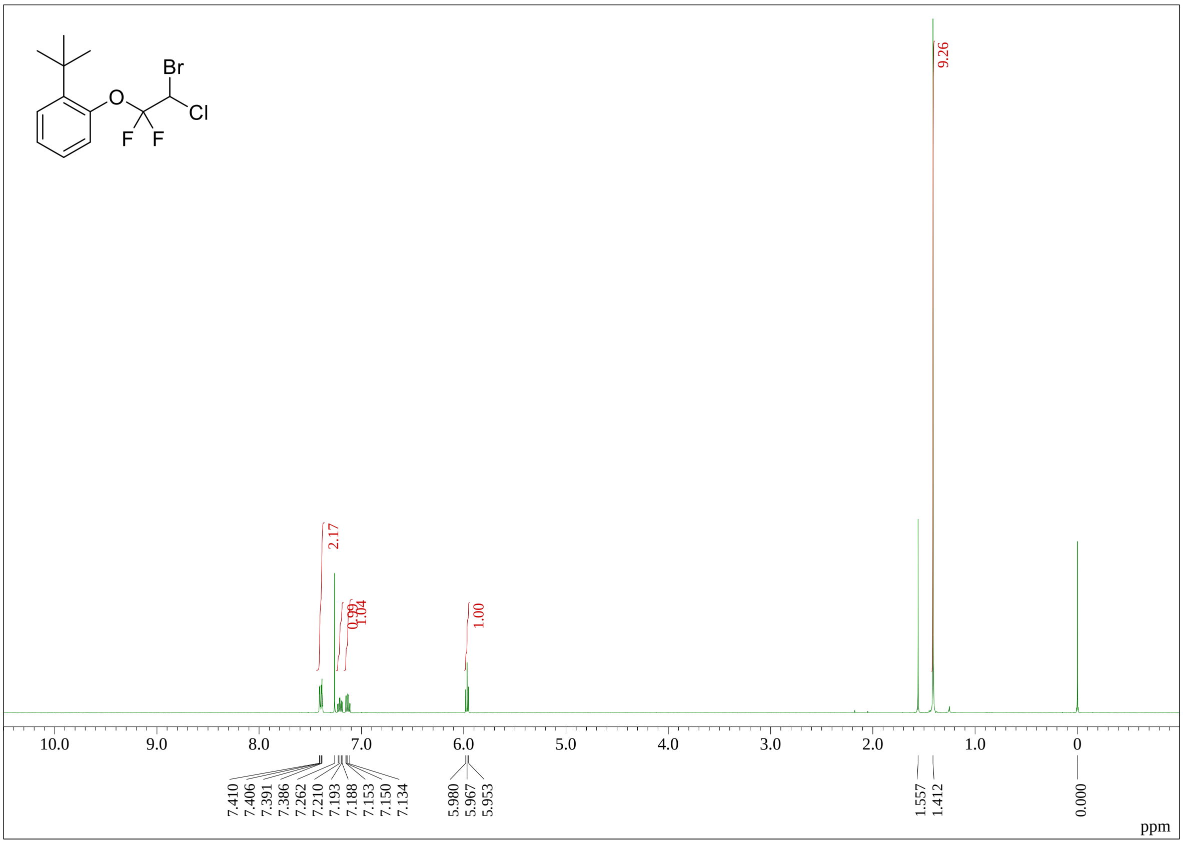

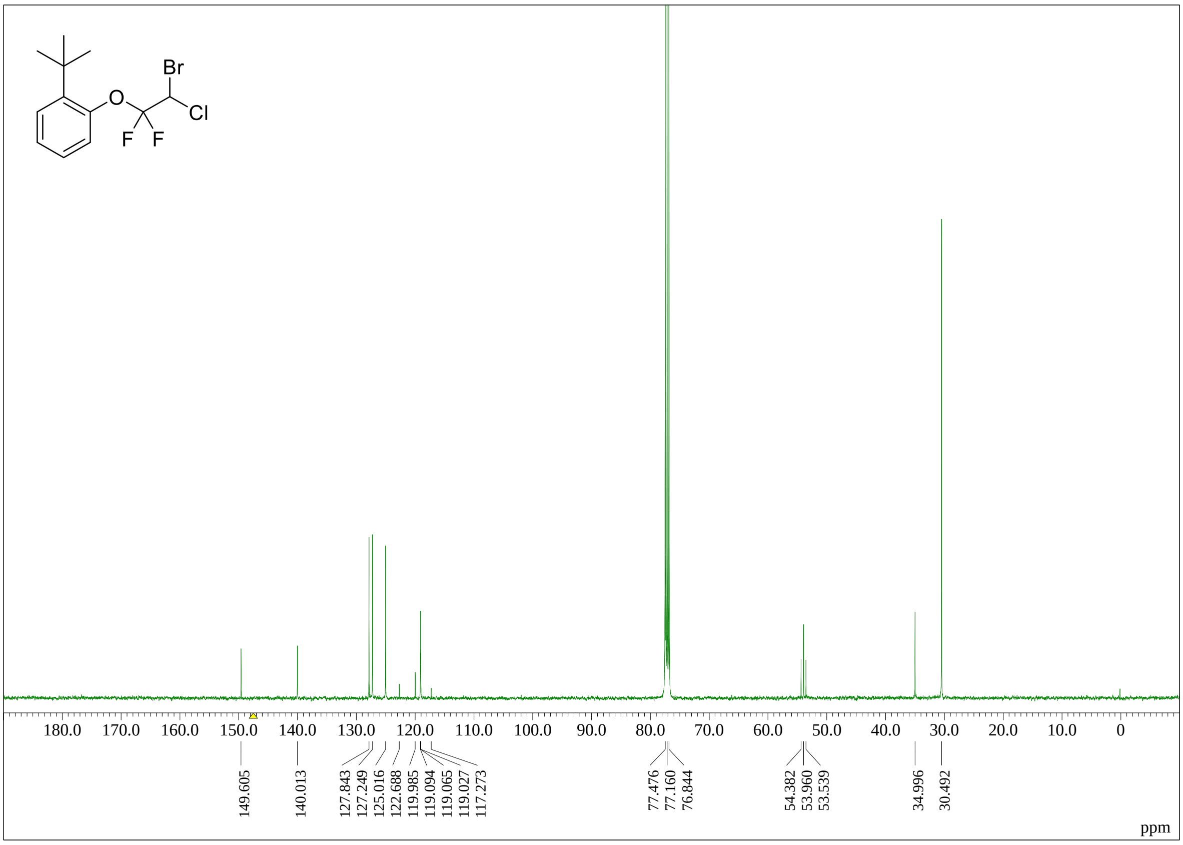

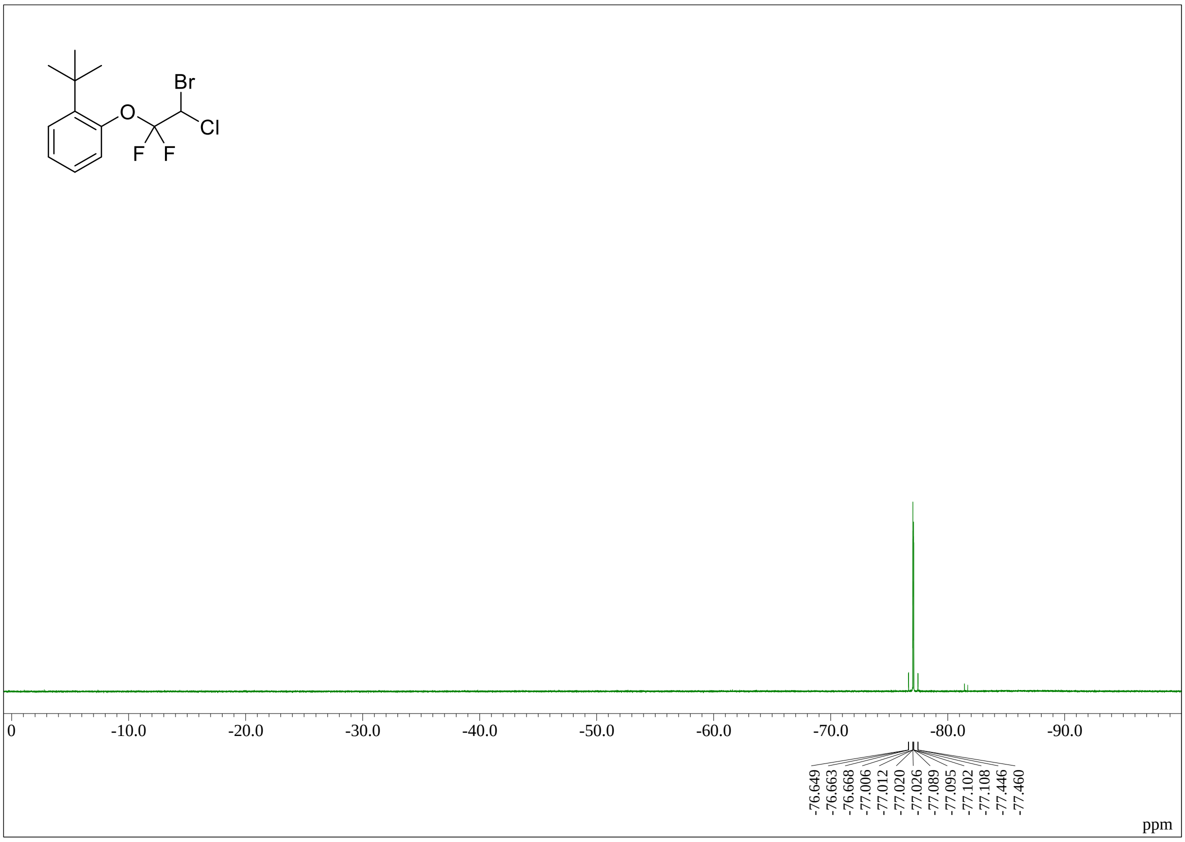

2-Bromo-2-chloro-1,1-difluoroethyl *p*-nitrophenyl ether (2d)

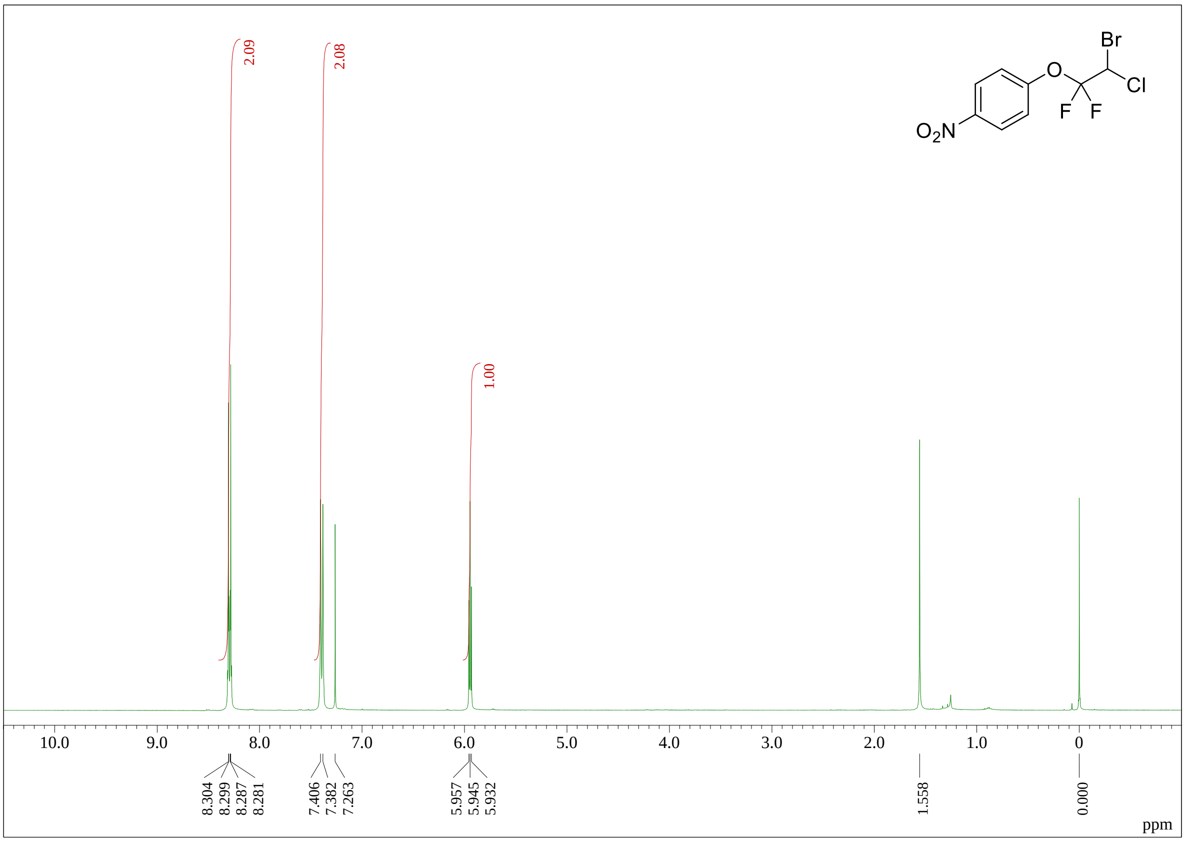

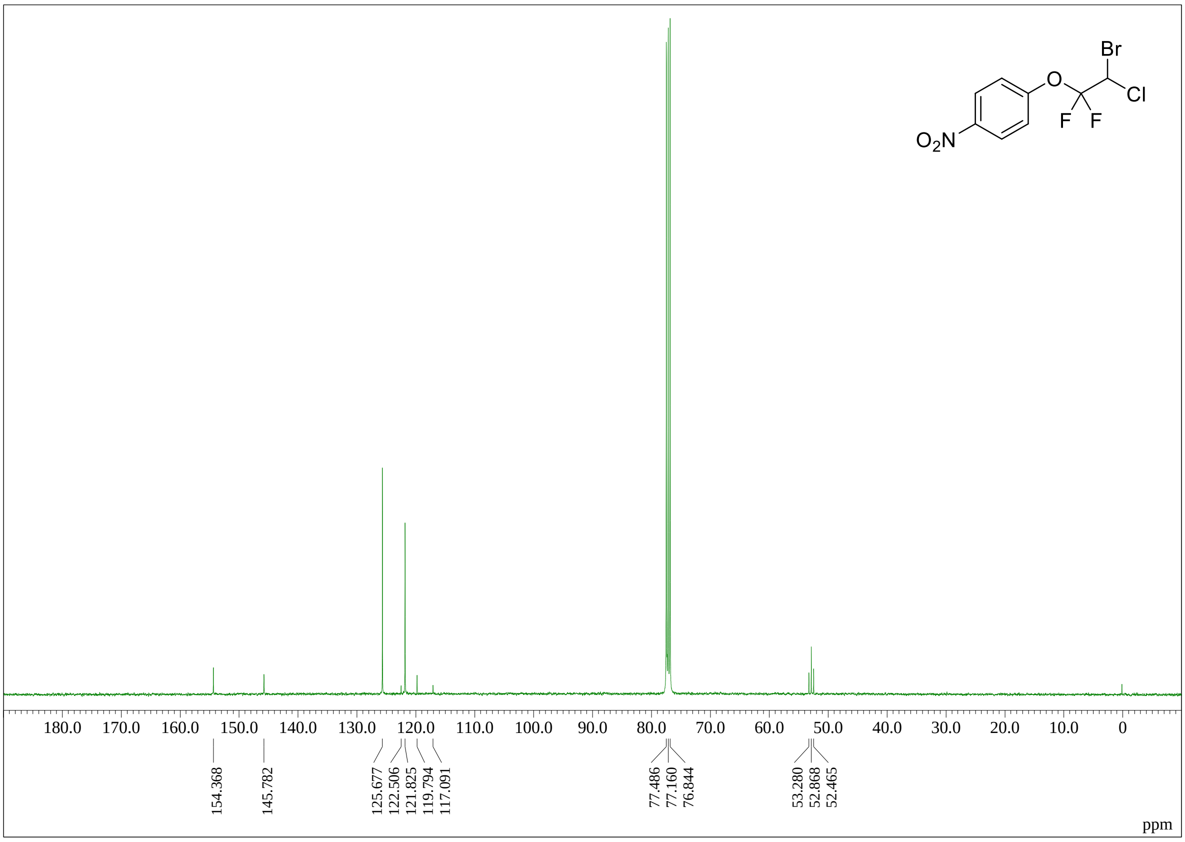
Br
O
Cl
F F
O2N
180.0 170.0 160.0 150.0 140.0 130.0 120.0 110.0 100.0 90.0 80.0 70.0 60.0 50.0 40.0 30.0 20.0 10.0 0
154.368
145.782
125.677
122.506
121.825
119.794
117.091
77.486
77.160
76.844
53.280
52.868
52.465
ppm

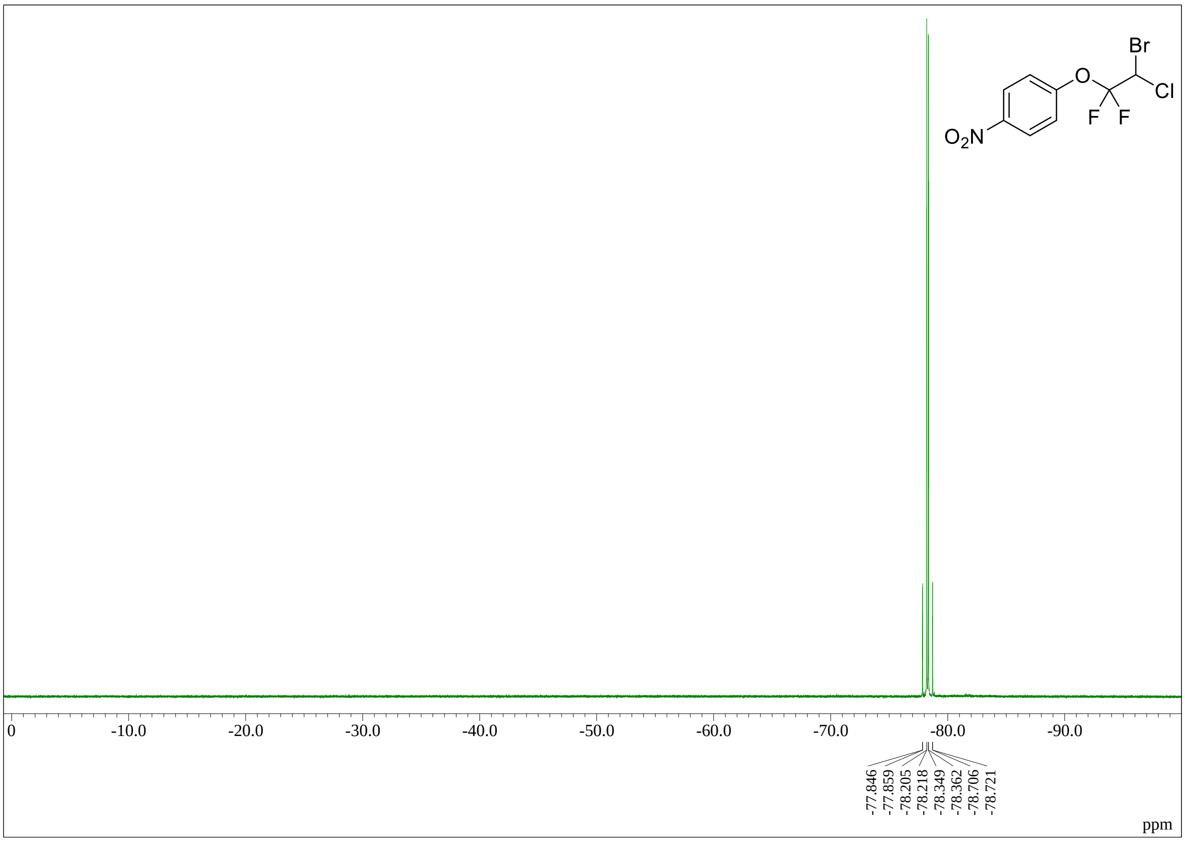
Br
O
Cl
F F
O2N
0 -10.0 -20.0 -30.0 -40.0 -50.0 -60.0 -70.0 -80.0 -90.0
-77.846
-77.859
-78.205
-78.218
-78.349
-78.362
-78.706
-78.721
ppm

2-Bromo-2-chloro-1,1-difluoroethyl *p*-trifluoromethylphenyl ether (2e)

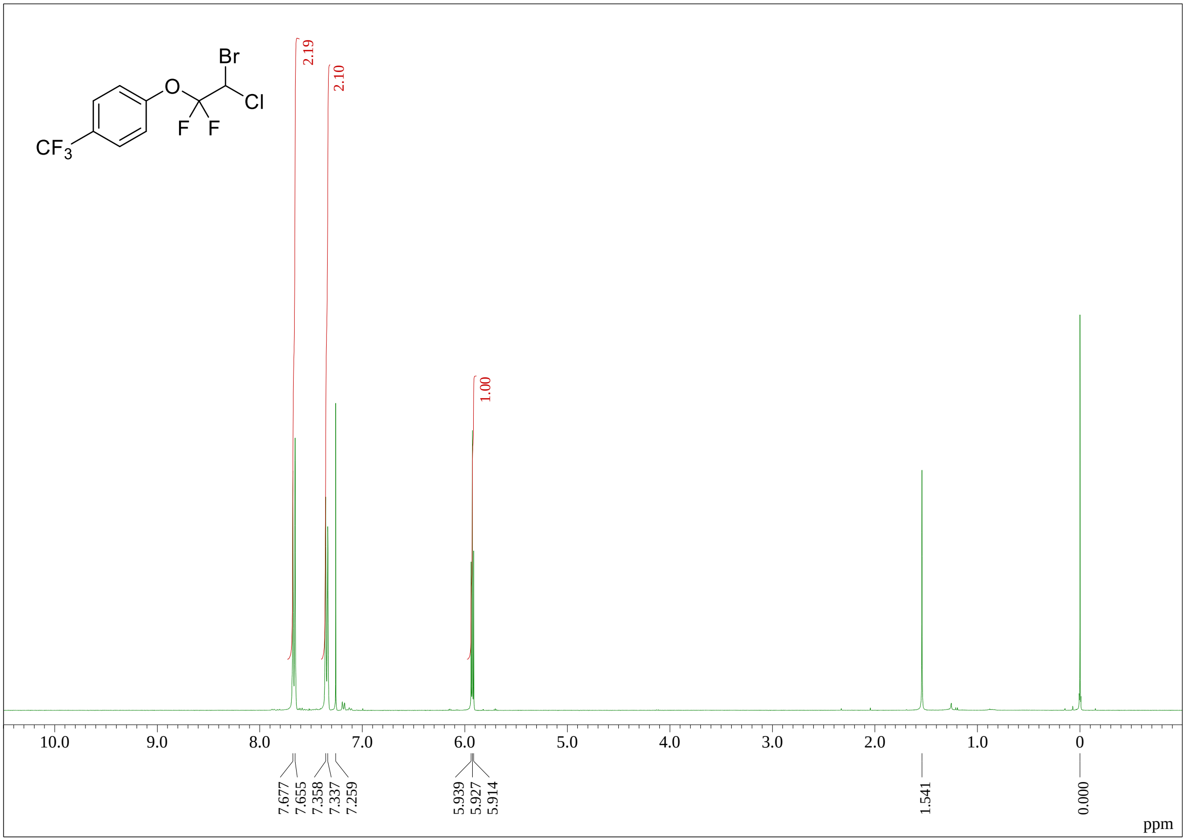

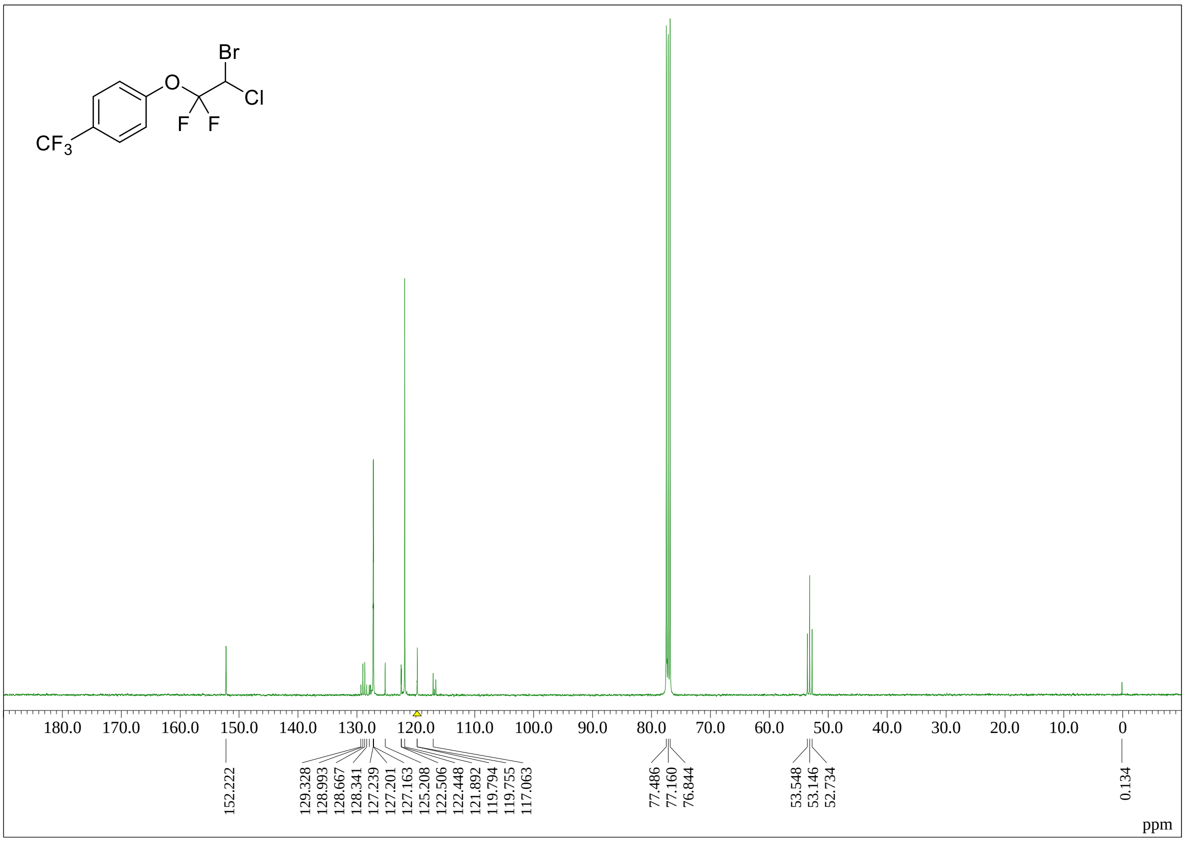

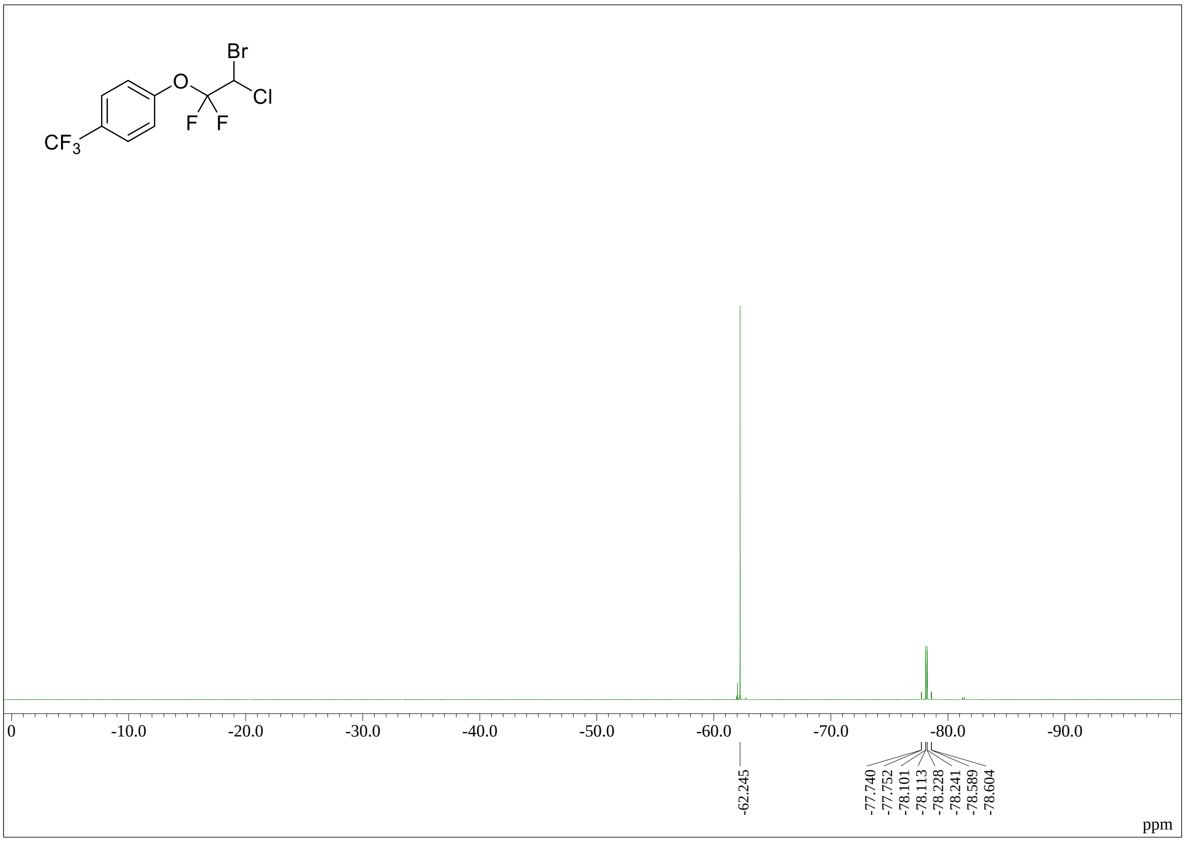

Ethyl *p*-(2-bromo-2-chloro-1,1-difluoroethoxy)benzoate (2f)

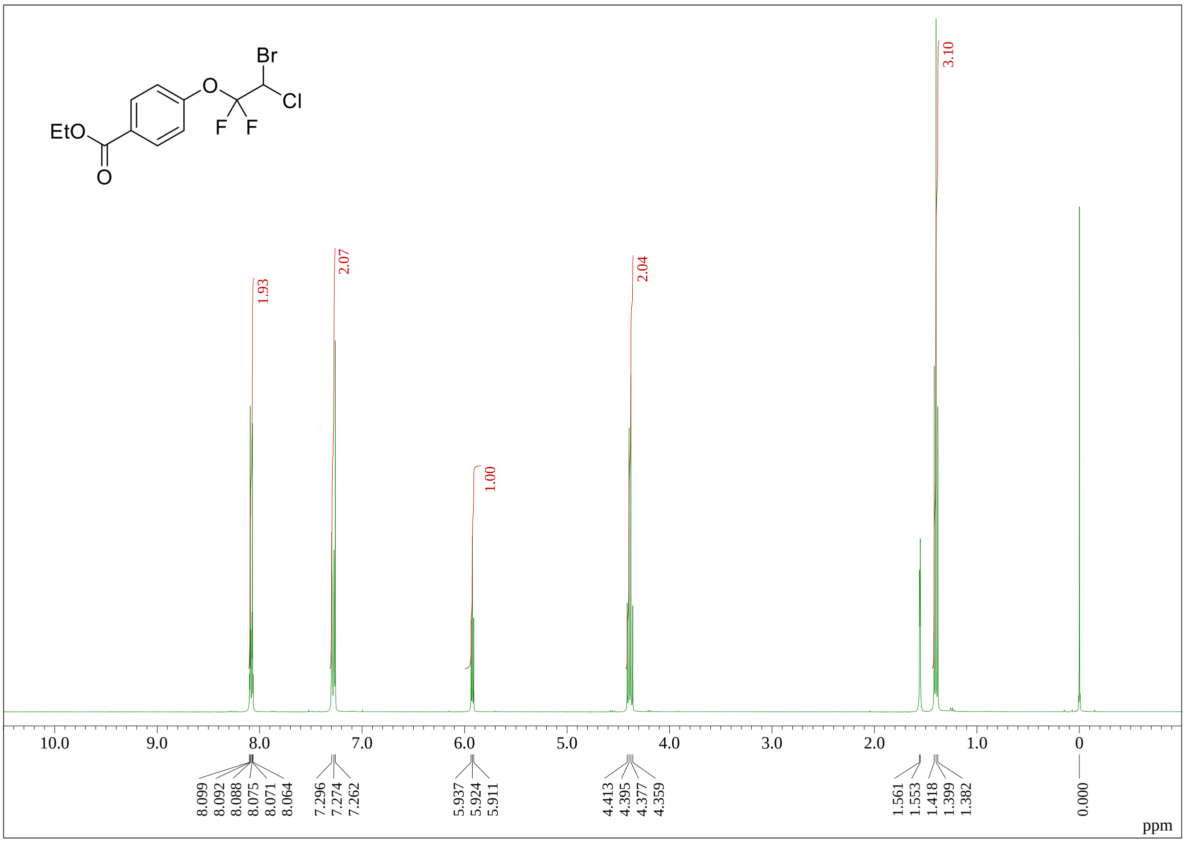

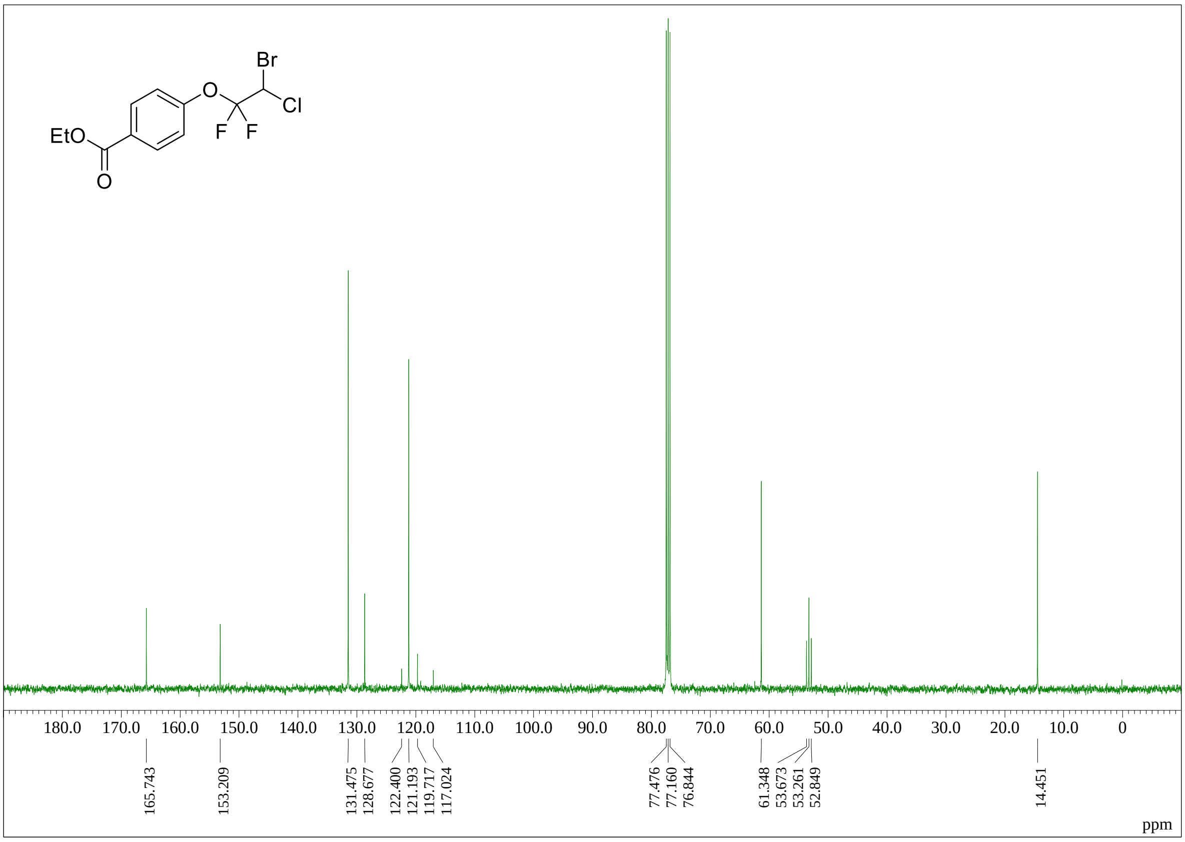
Br
Cl
O
EtO
F F
O
180.0 170.0 160.0 150.0 140.0 130.0 120.0 110.0 100.0 90.0 80.0 70.0 60.0 50.0 40.0 30.0 20.0 10.0 0
165.743
153.209
131.475
128.677
122.400
121.193
119.717
117.024
77.476
77.160
76.844
61.348
53.673
53.261
52.849
14.451
ppm

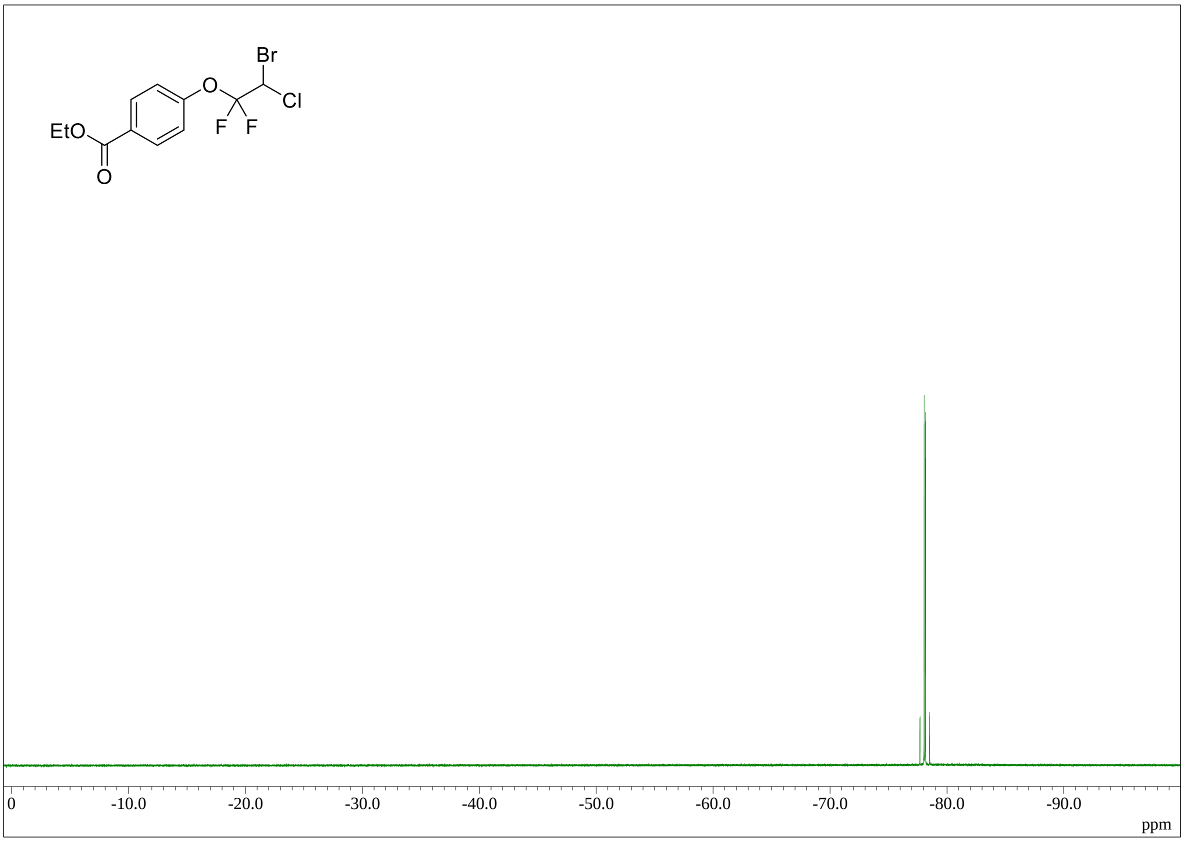
Br
Cl
O
EtO
F F
O
0 -10.0 -20.0 -30.0 -40.0 -50.0 -60.0 -70.0 -80.0 -90.0
ppm

2-Bromo-2-chloro-1,1-difluoroethyl *o*-phenylphenyl ether (2h)

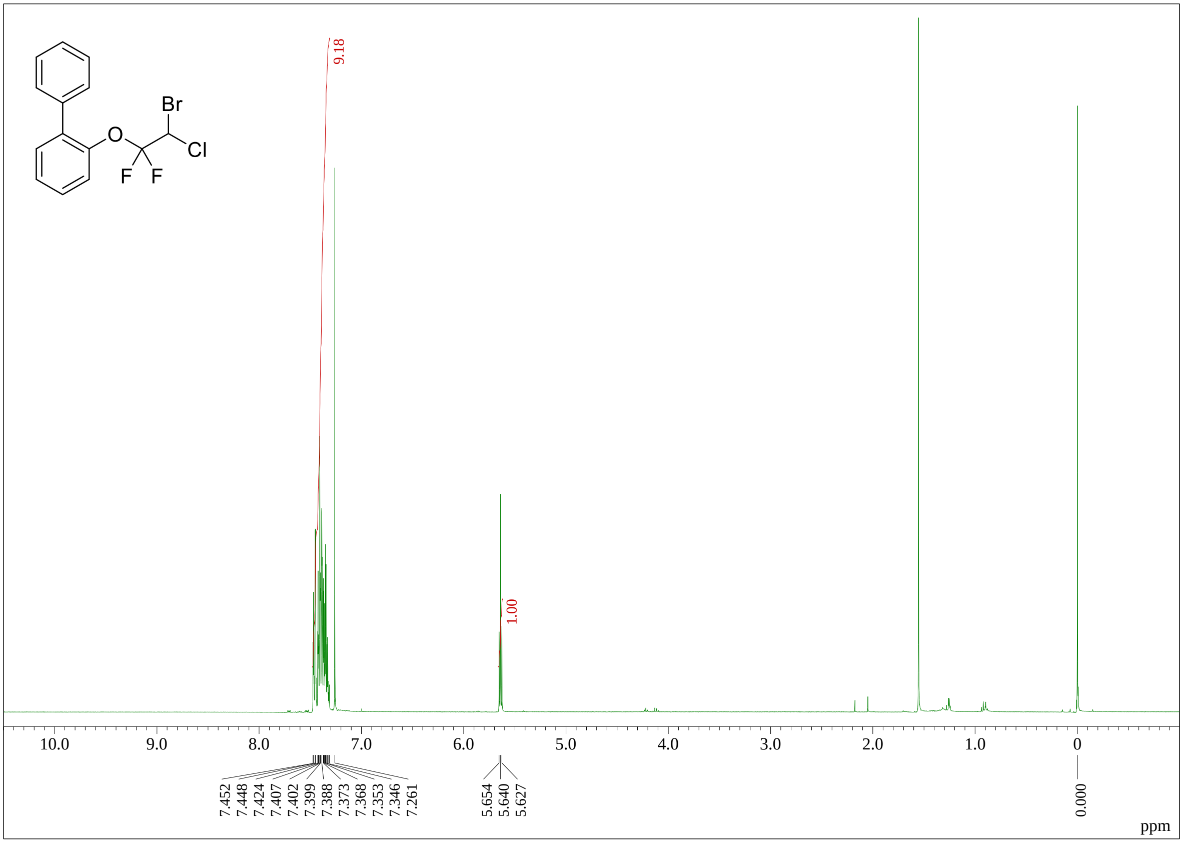

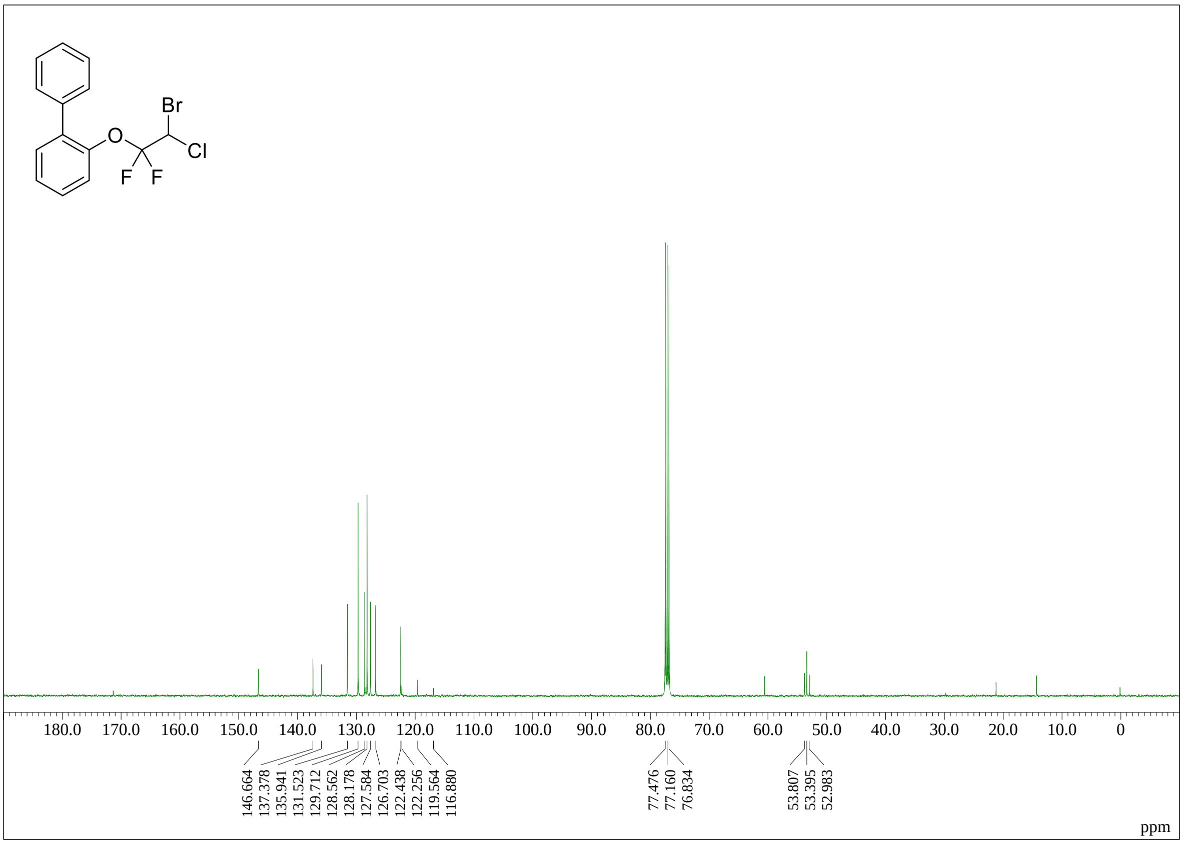

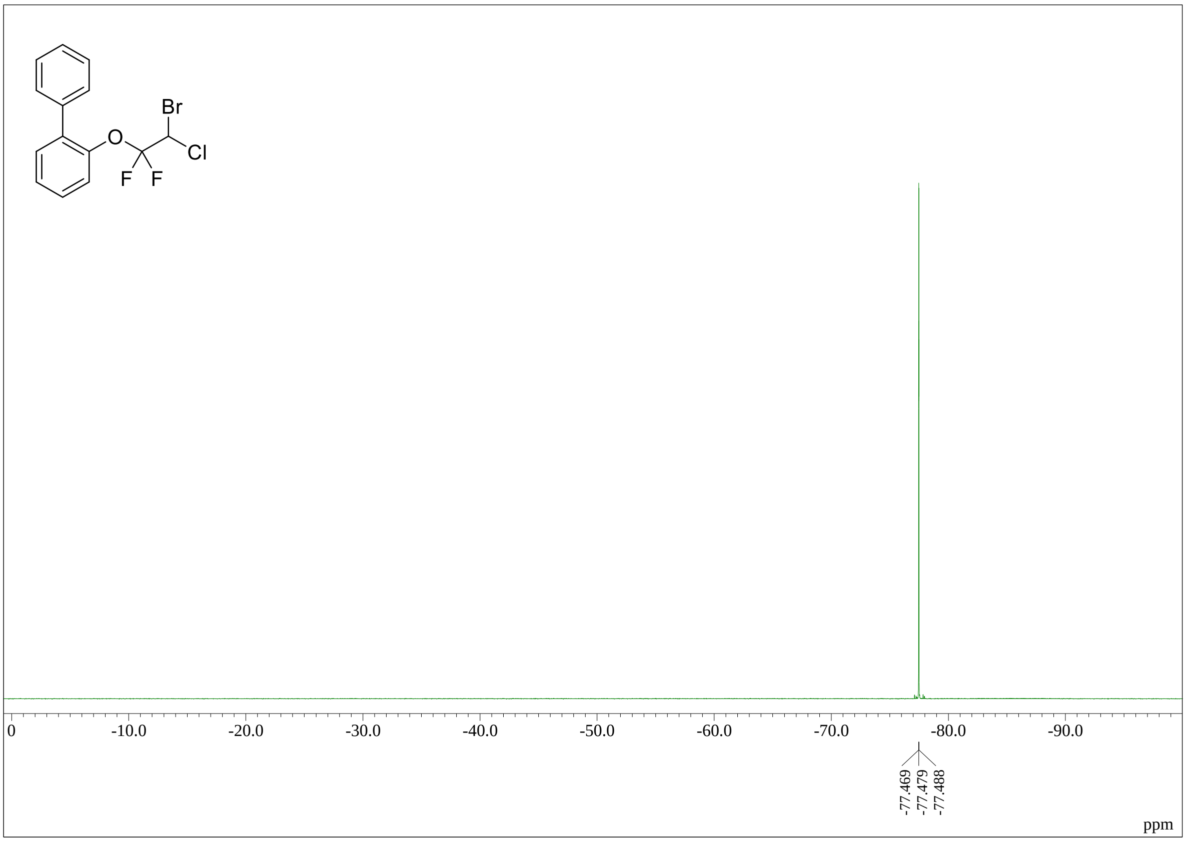

2-Bromo-2-chloro-1,1-difluoroethyl *m*-phenylphenyl ether (2i)

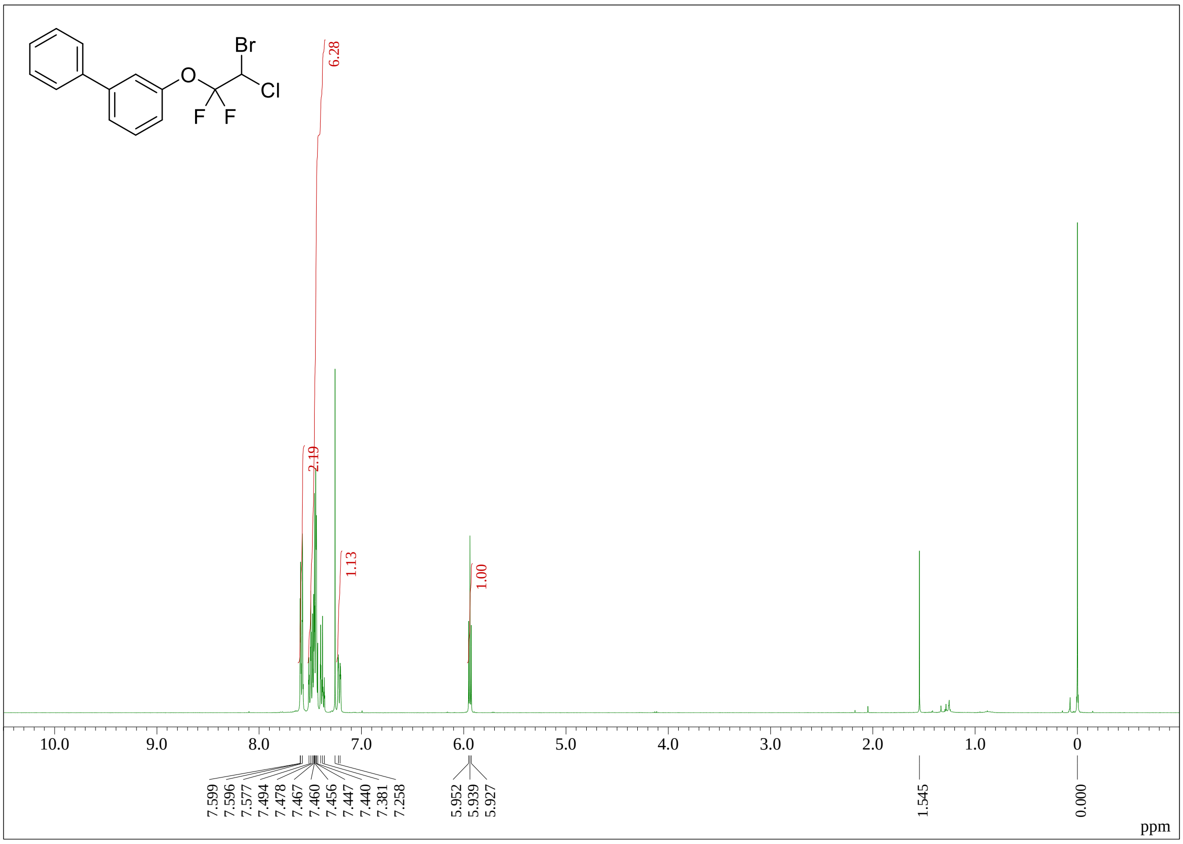

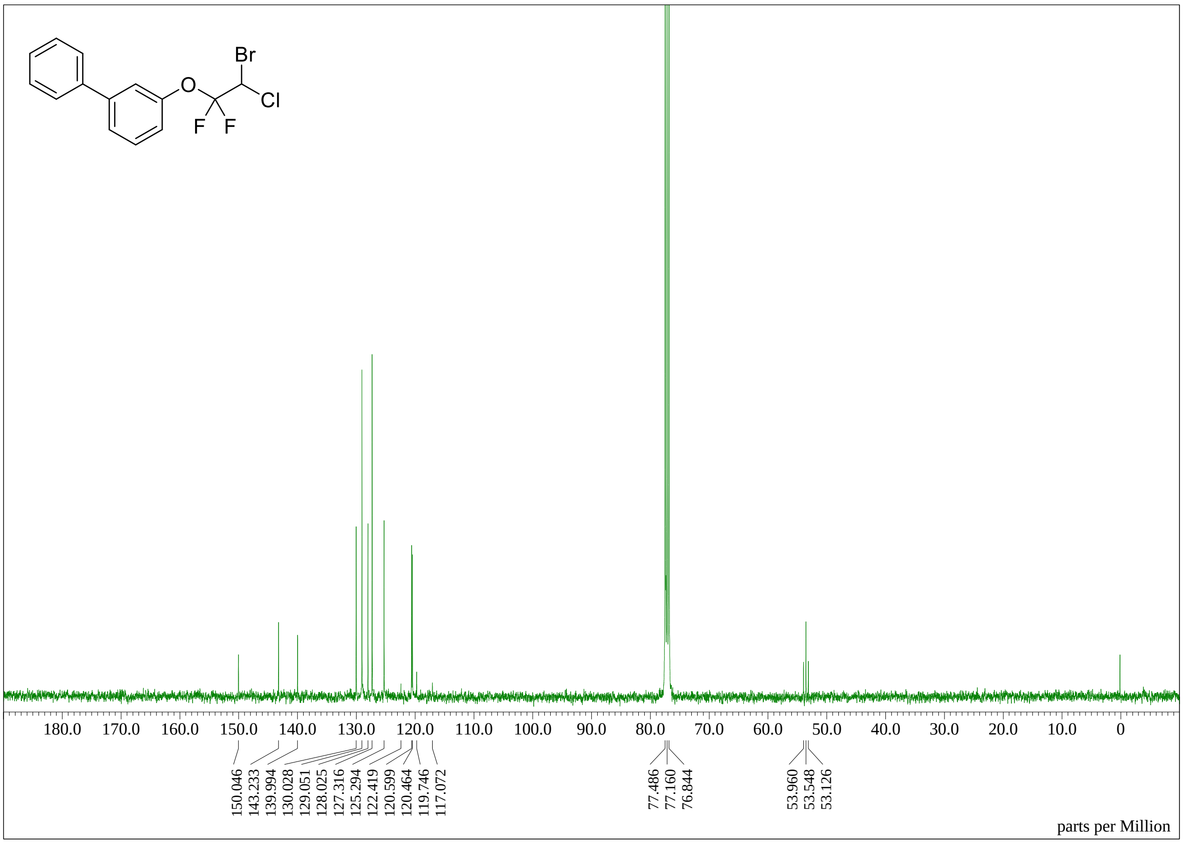
Br
O
Cl
F F
180.0 170.0 160.0 150.0 140.0 130.0 120.0 110.0 100.0 90.0 80.0 70.0 60.0 50.0 40.0 30.0 20.0 10.0 0
150.046
143.233
139.994
130.028
129.051
128.025
127.316
125.294
122.419
120.599
120.464
119.746
117.072
77.486
77.160
76.844
53.960
53.548
53.126
parts per Million

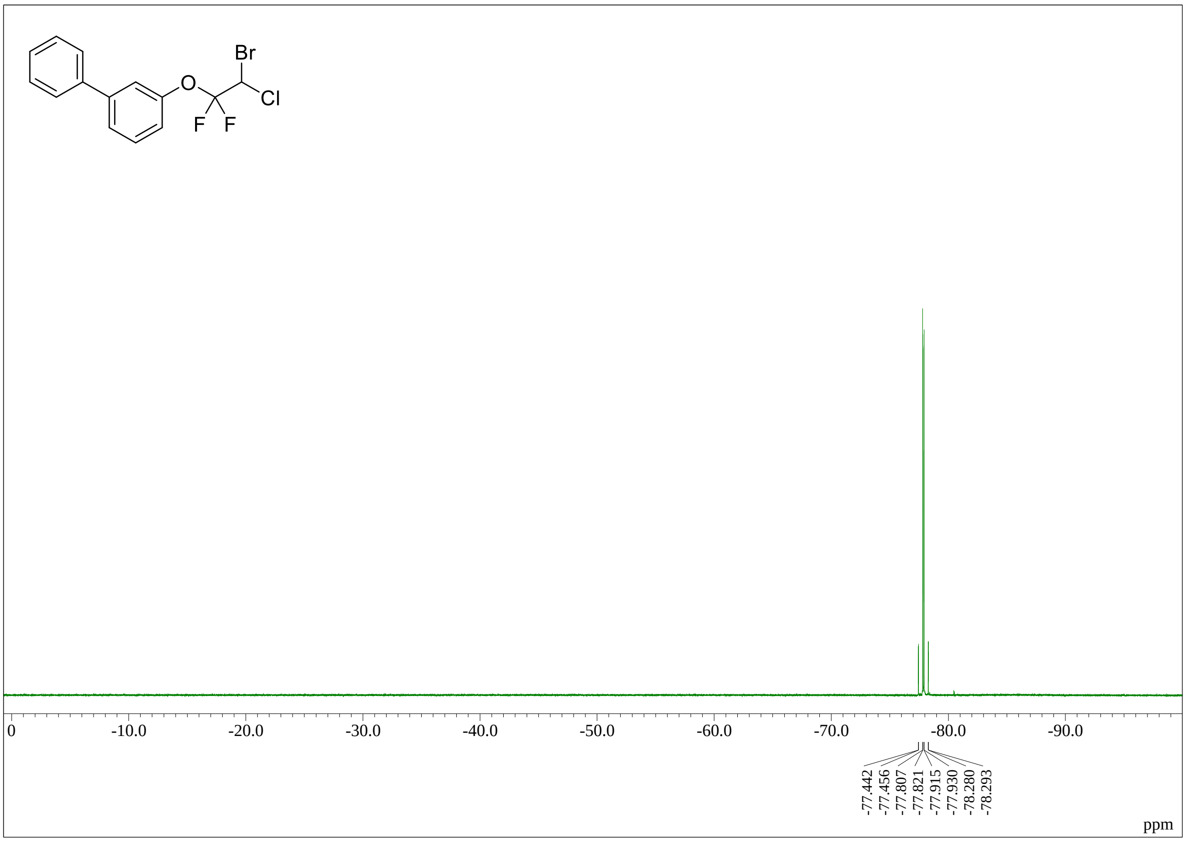
Br
O
Cl
F F
0 -10.0 -20.0 -30.0 -40.0 -50.0 -60.0 -70.0 -80.0 -90.0
-77.442
-77.456
-77.807
-77.821
-77.915
-77.930
-78.280
-78.293
ppm

2-Bromo-2-chloro-1,1-difluoroethyl *p*-phenylphenyl ether (2j)

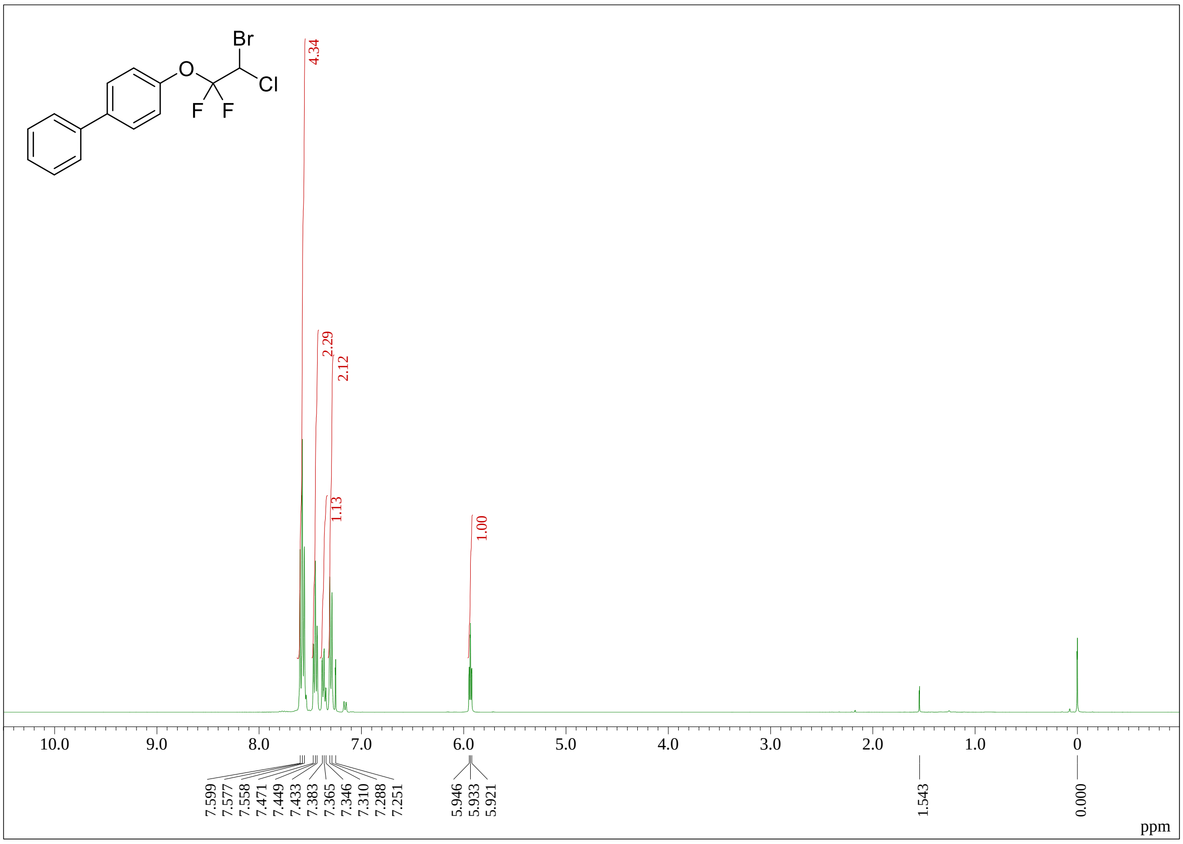

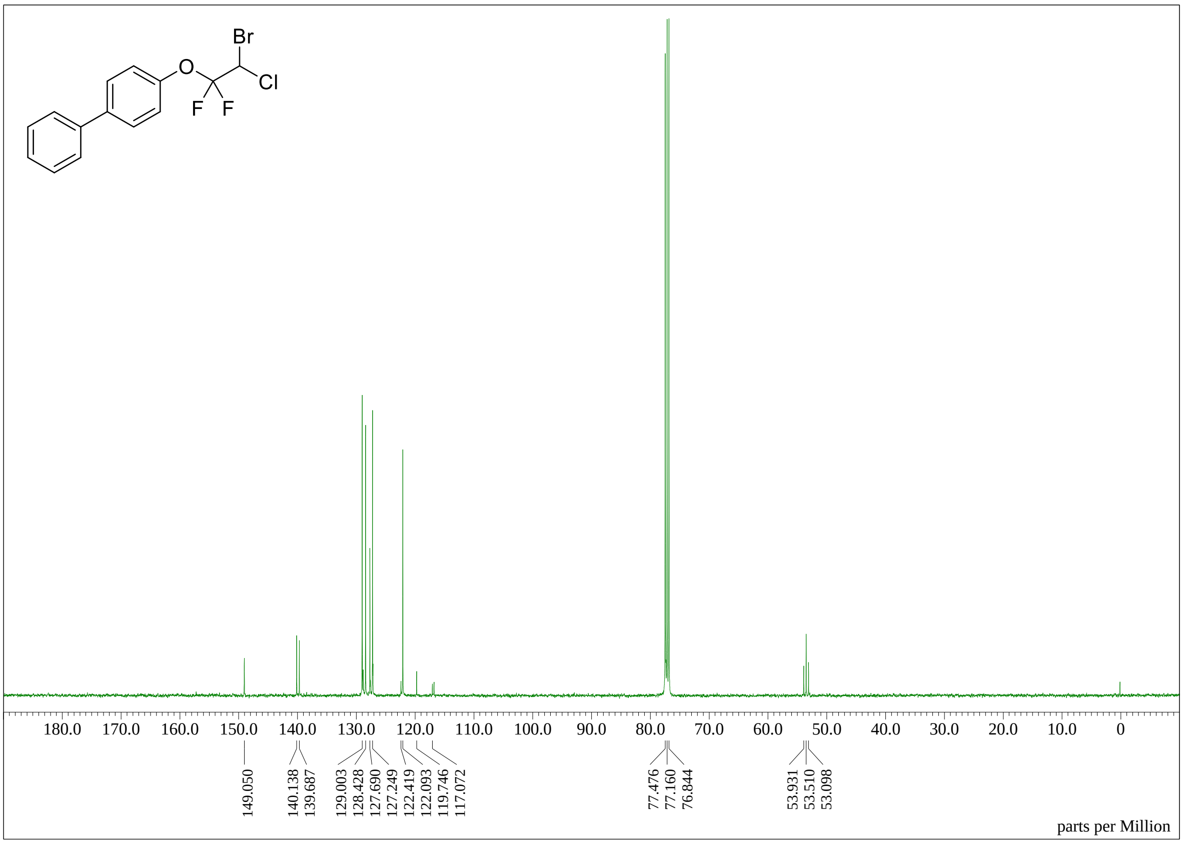

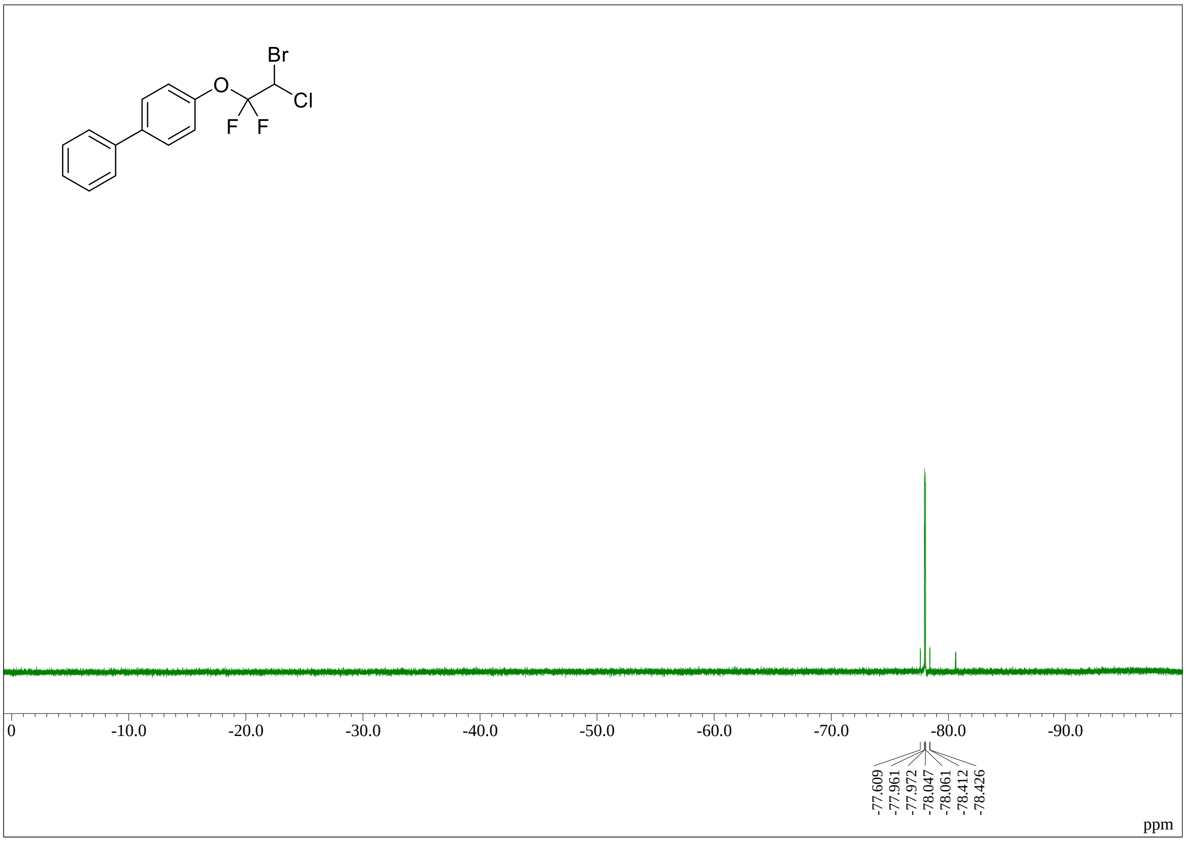

2-Bromo-2-chloro-1,1-difluoroethyl 1-naphthyl ether (2k)

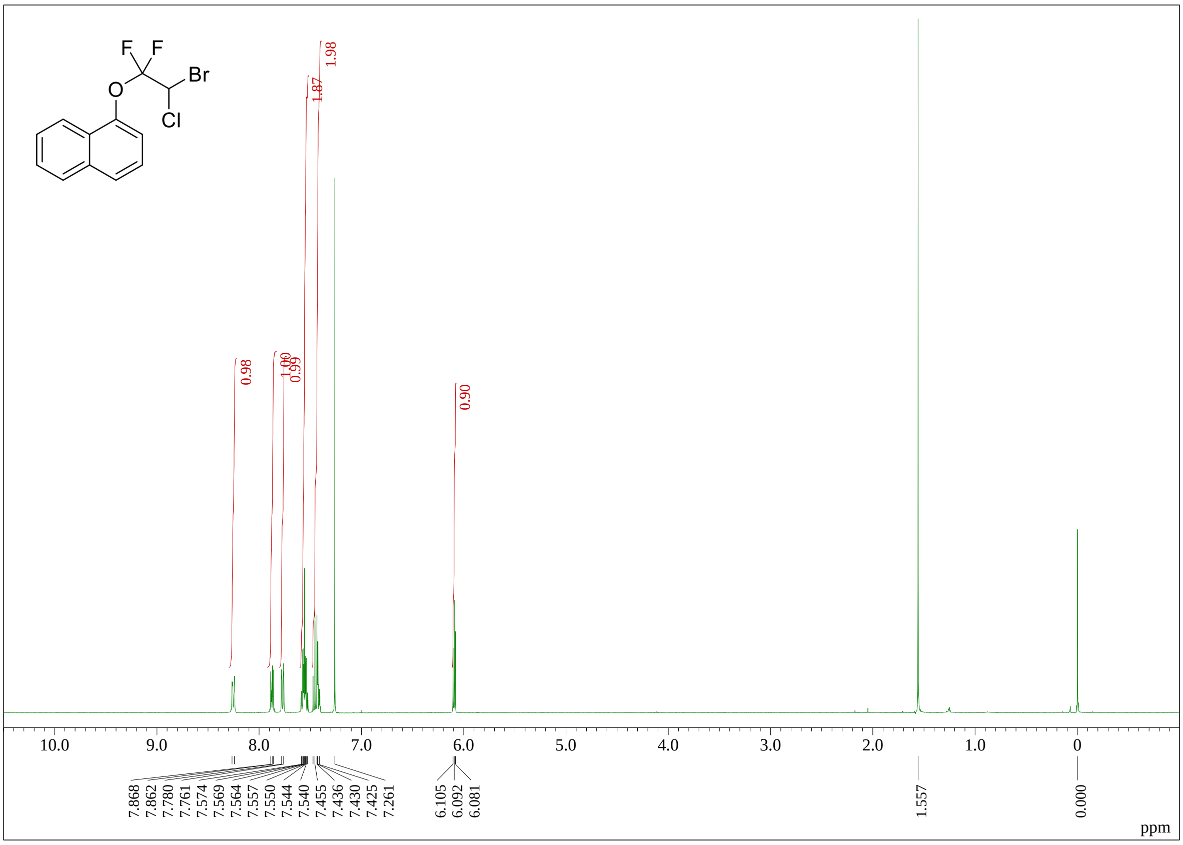

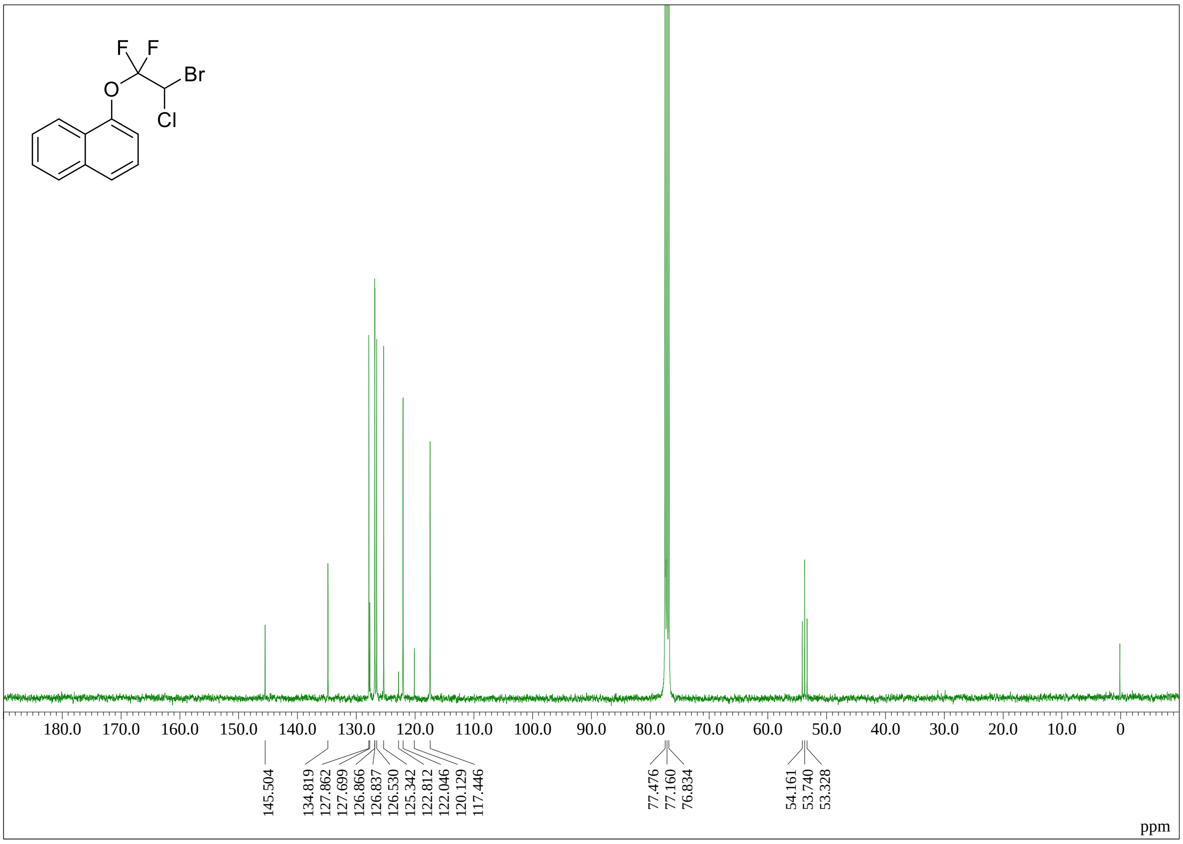
F F
Br
O
Cl
180.0 170.0 160.0 150.0 140.0 130.0 120.0 110.0 100.0 90.0 80.0 70.0 60.0 50.0 40.0 30.0 20.0 10.0 0
145.504
134.819
127.862
127.699
126.866
126.837
126.530
125.342
122.812
122.046
120.129
117.446
77.476
77.160
76.834
54.161
53.740
53.328
ppm

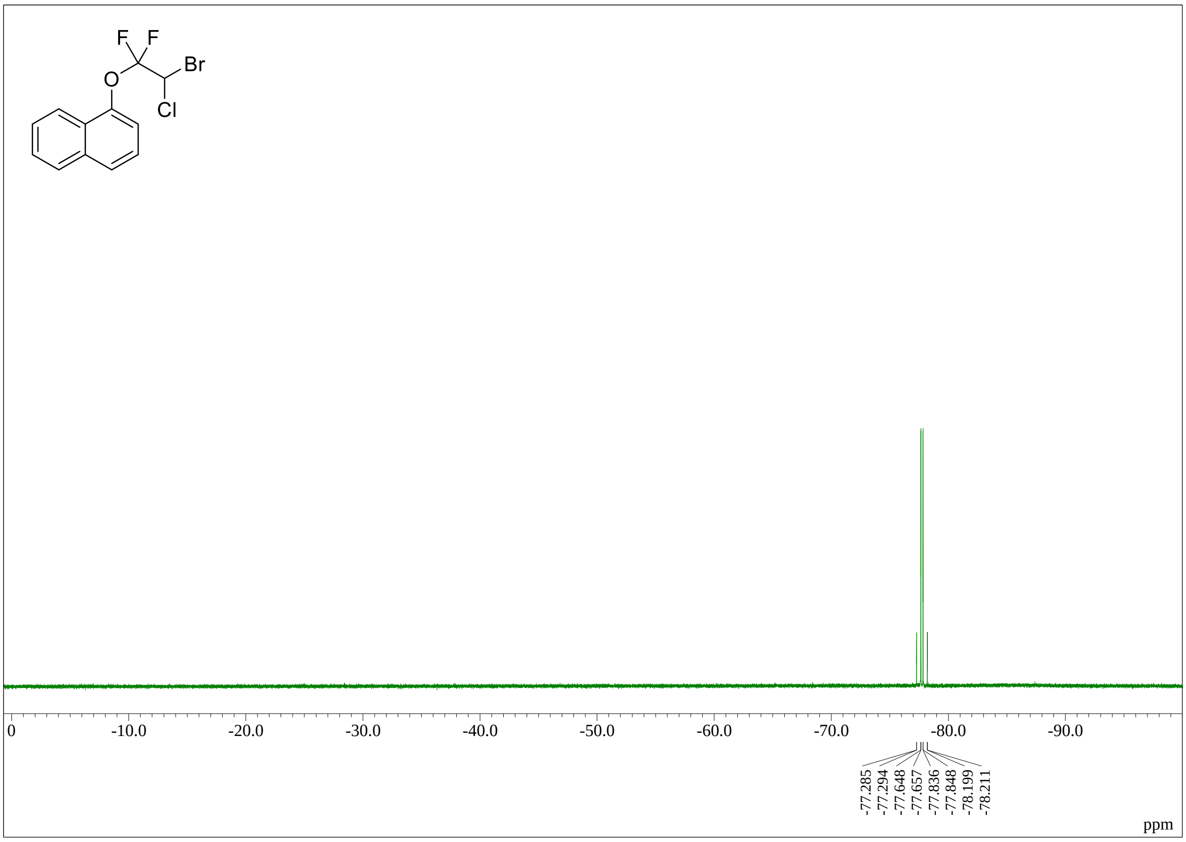
F F
Br
O
Cl
0 -10.0 -20.0 -30.0 -40.0 -50.0 -60.0 -70.0 -80.0 -90.0
-77.285
-77.294
-77.648
-77.657
-77.836
-77.848
-78.199
-78.211
ppm

2-Bromo-2-chloro-1,1-difluoroethyl *o*-iodophenyl ether (2l)

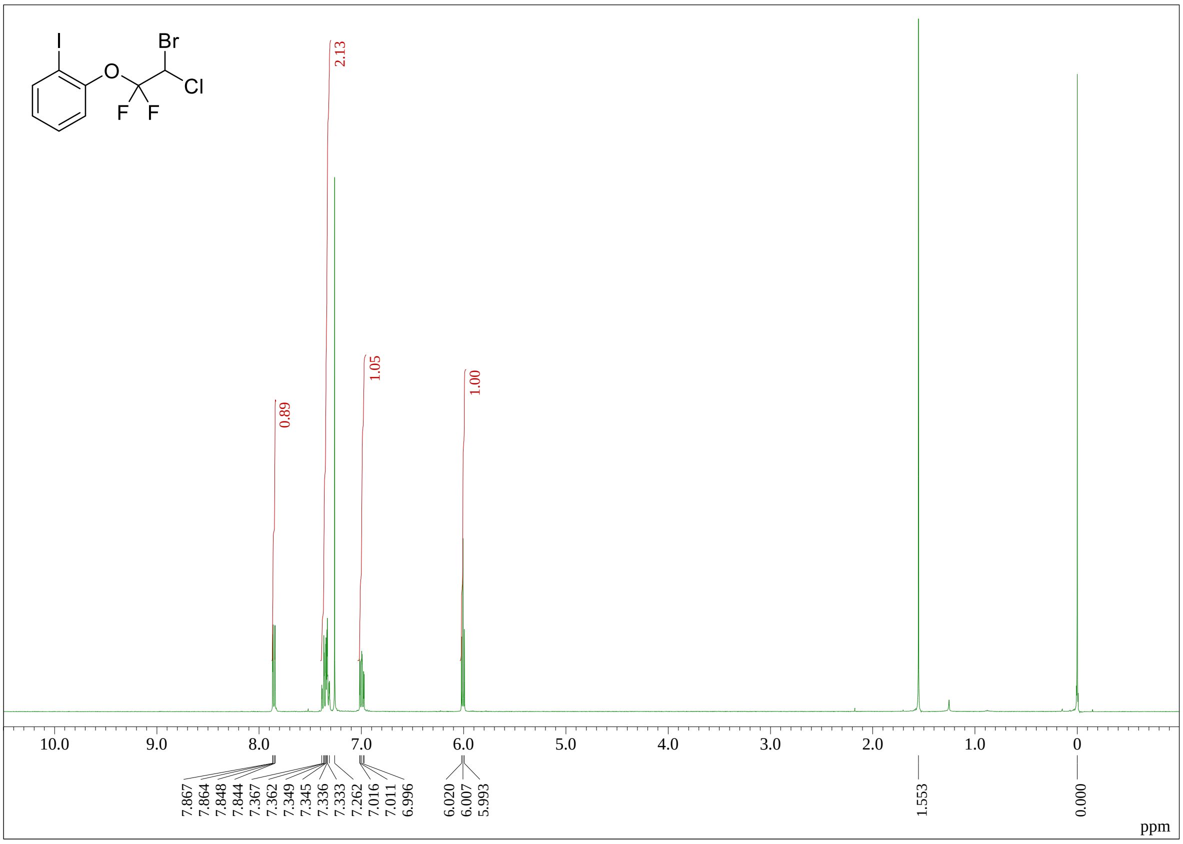

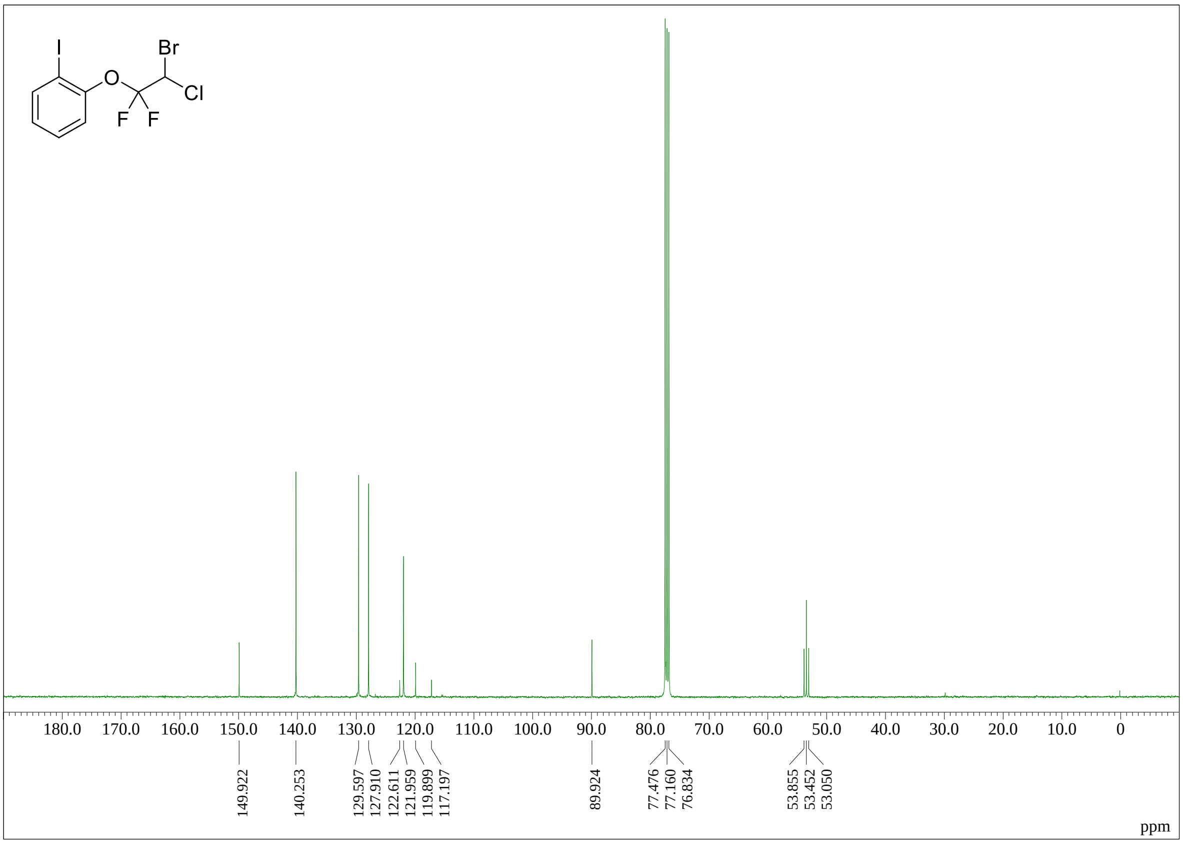

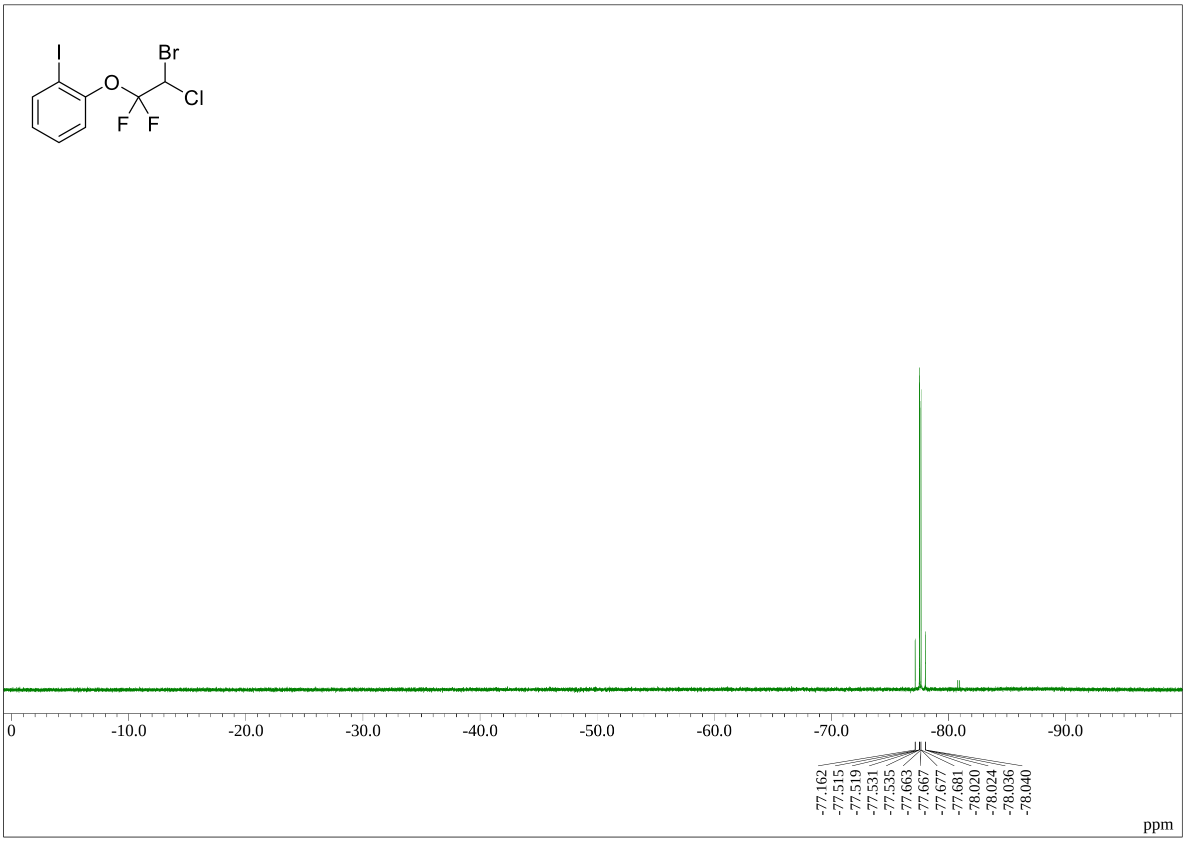

o-Allylphenyl 2-bromo-2-chloro-1,1-difluoroethyl ether (2m)

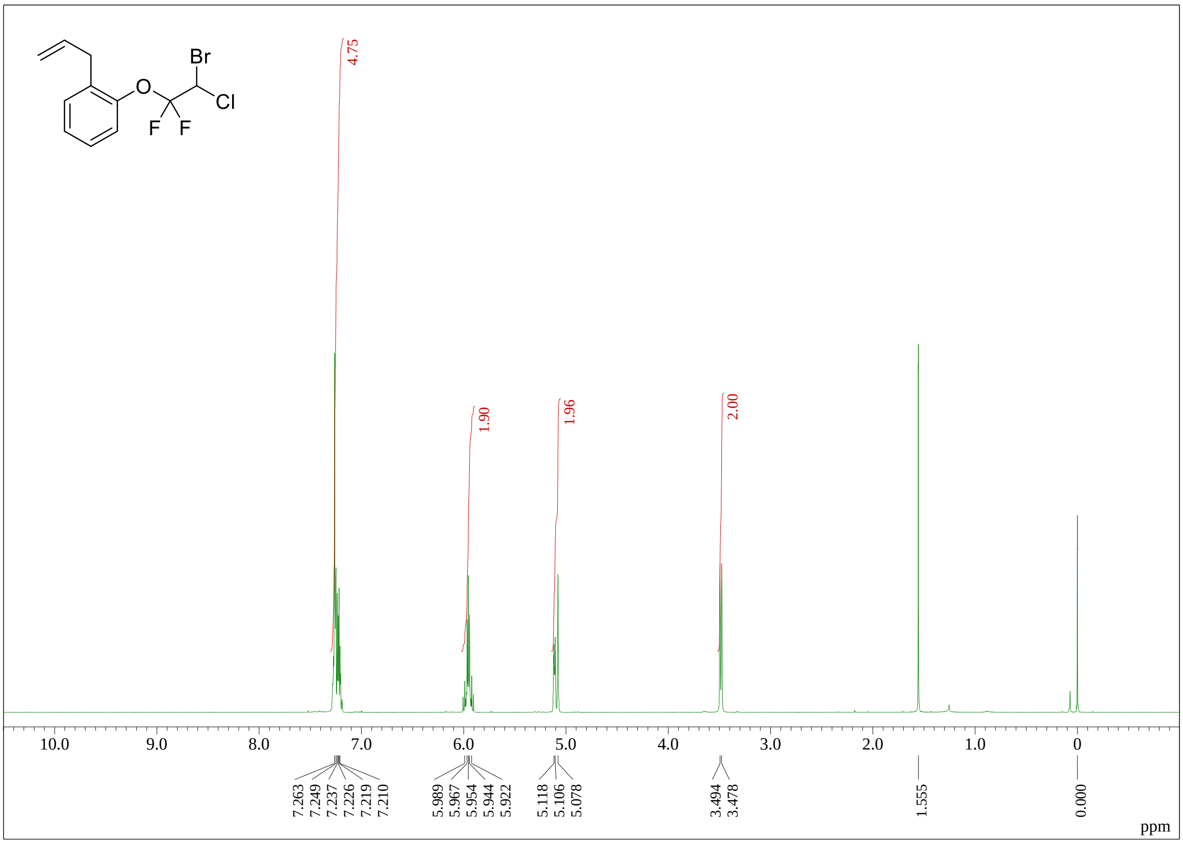

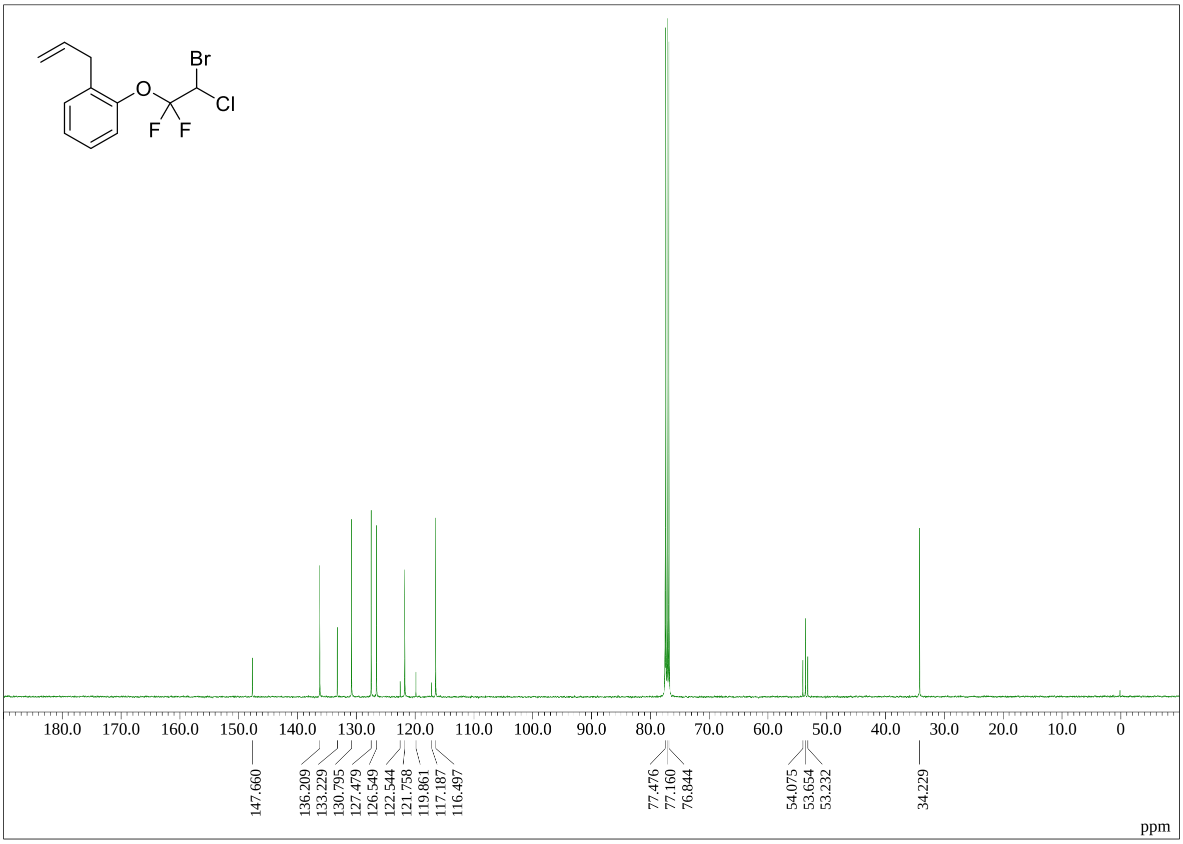
Br
O
Cl
F F
180.0 170.0 160.0 150.0 140.0 130.0 120.0 110.0 100.0 90.0 80.0 70.0 60.0 50.0 40.0 30.0 20.0 10.0 0
147.660
136.209
133.229
130.795
127.479
126.549
122.544
121.758
119.861
117.187
116.497
77.476
77.160
76.844
54.075
53.654
53.232
34.229
ppm

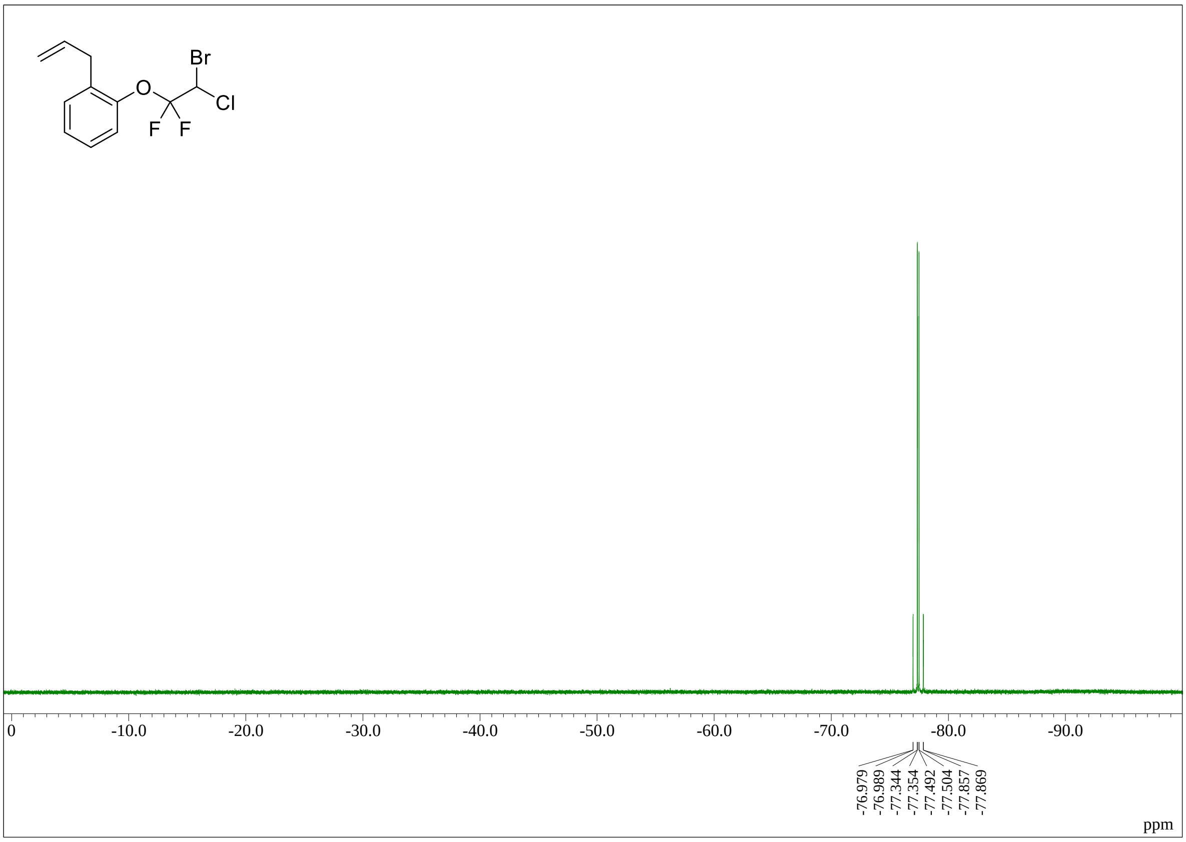
Br
O
Cl
F F
0 -10.0 -20.0 -30.0 -40.0 -50.0 -60.0 -70.0 -80.0 -90.0
-76.979
-76.989
-77.344
-77.354
-77.492
-77.504
-77.857
-77.869
ppm

2-Bromo-2-chloro-1,1-difluoroethyl *o*-propenylphenyl ether (2n)

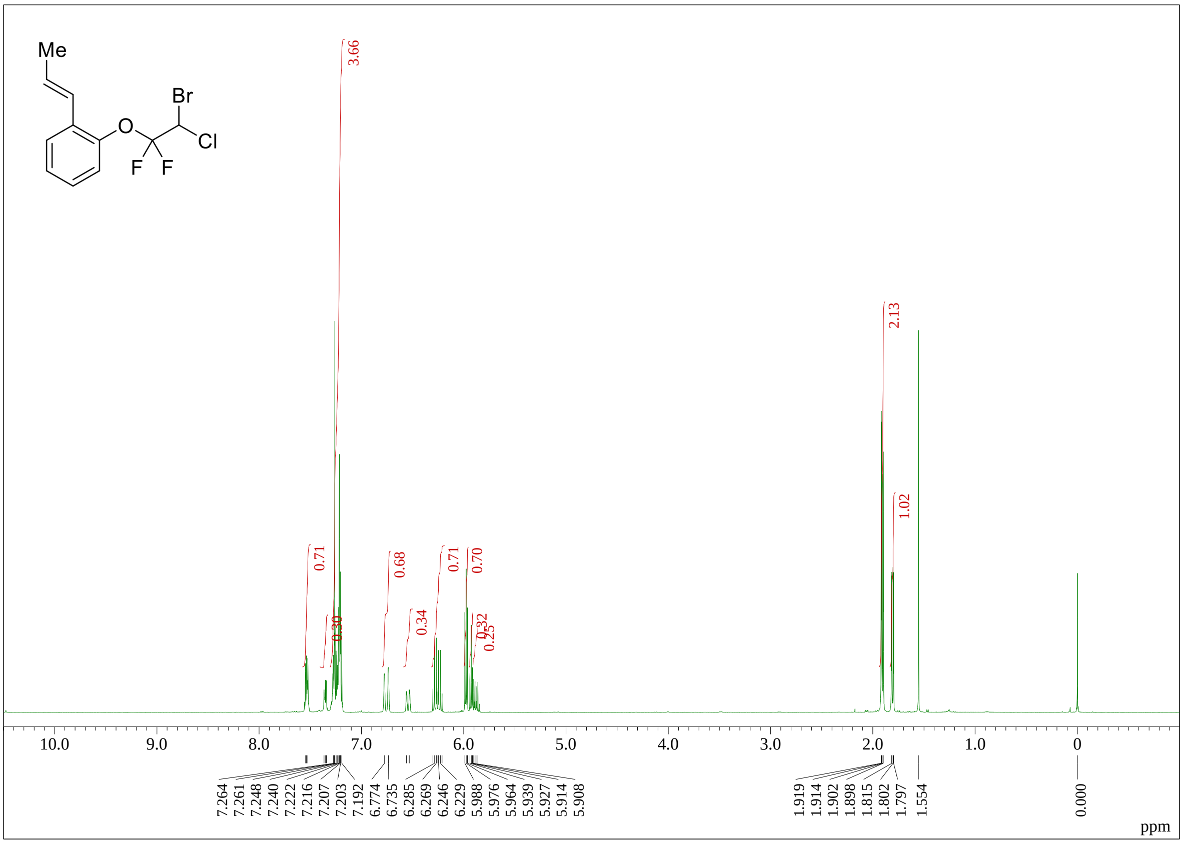

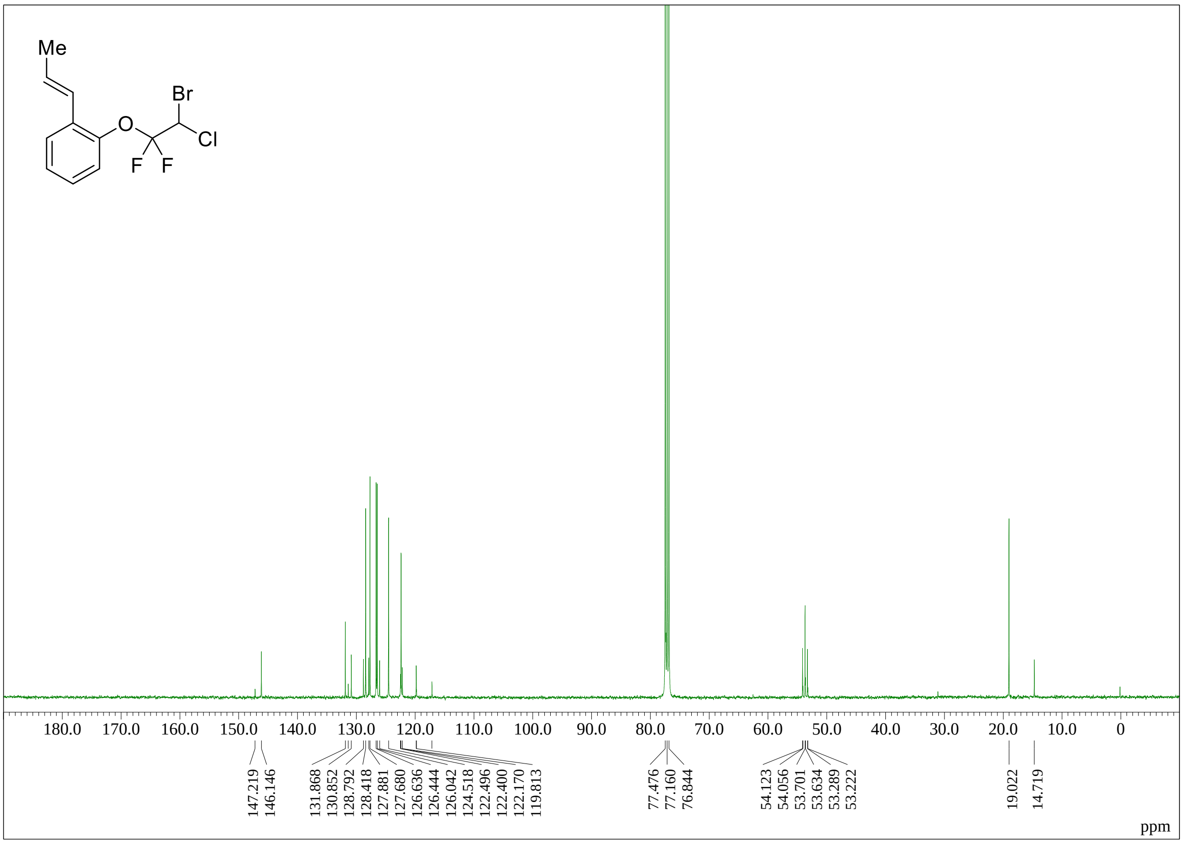

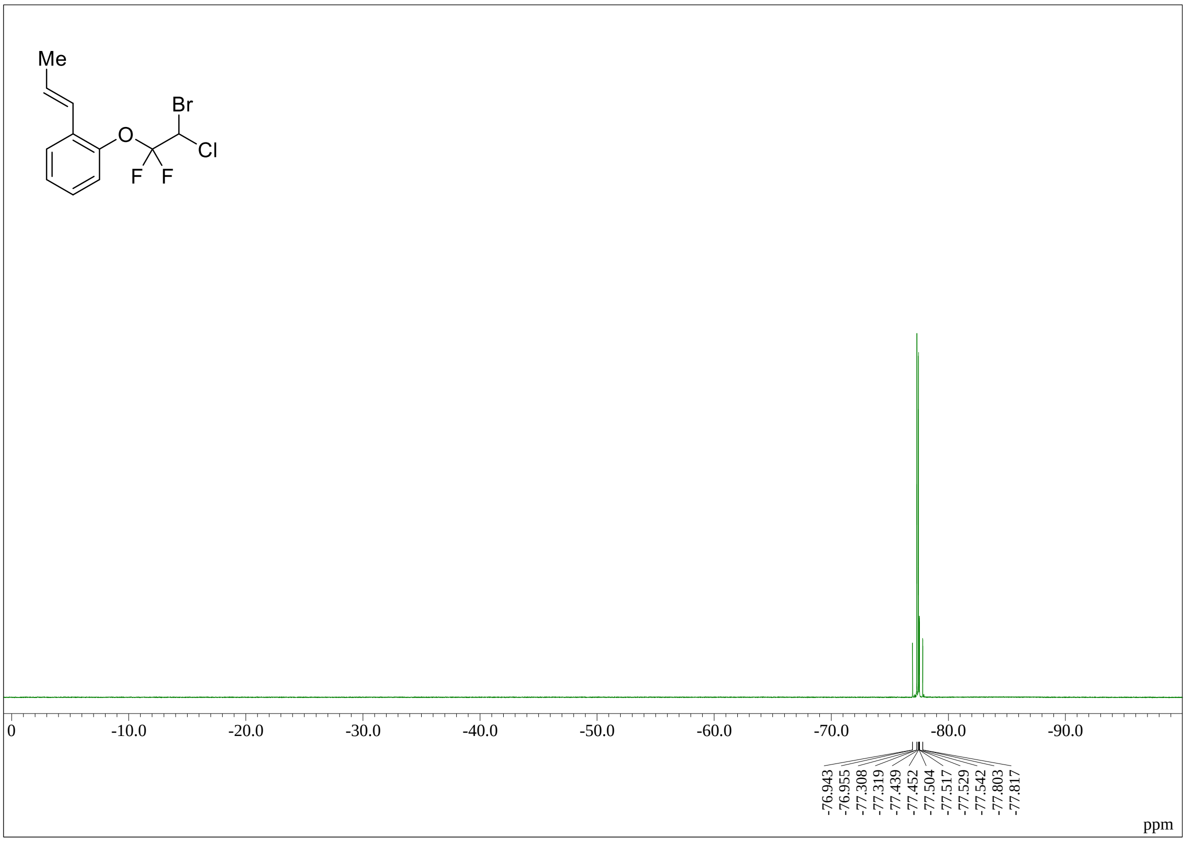

(*E*)-2'-(2-Bromo-2-chloro-1,1-difluoroethoxy)-chalcone (2o)

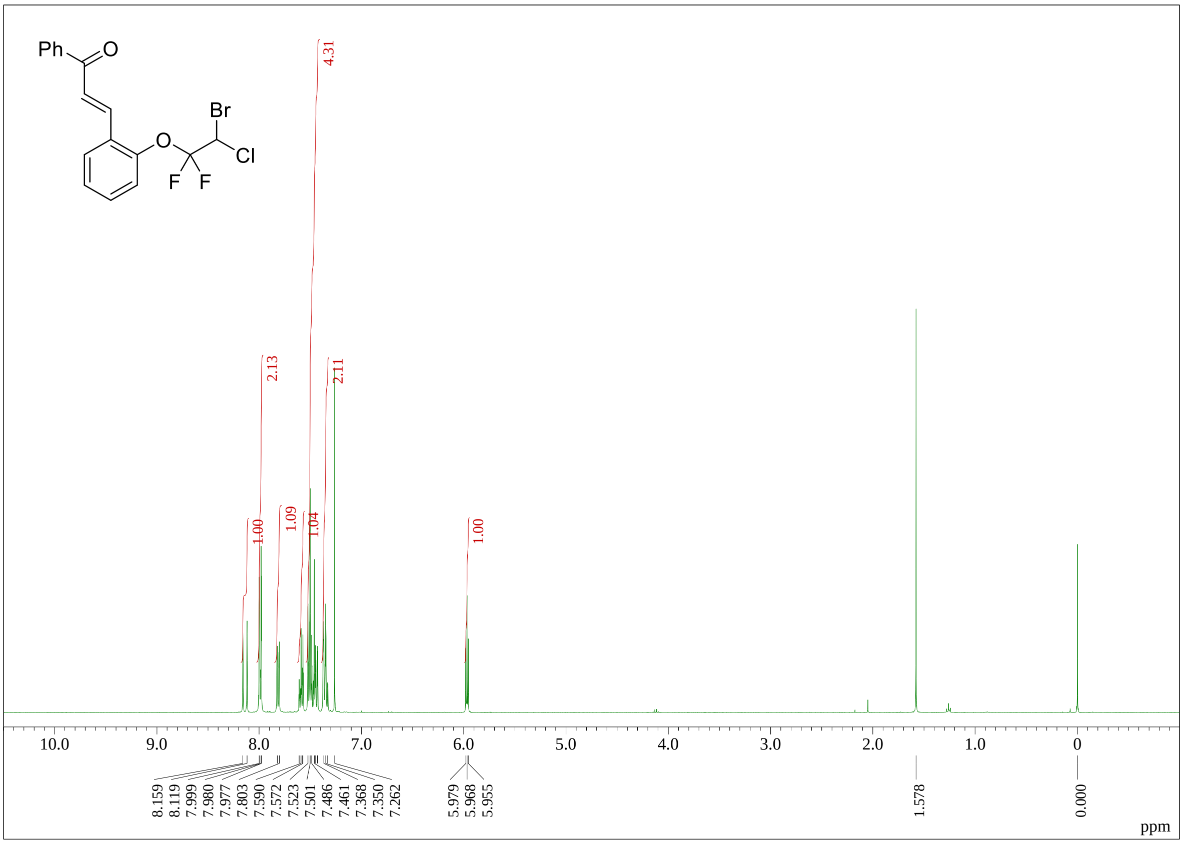

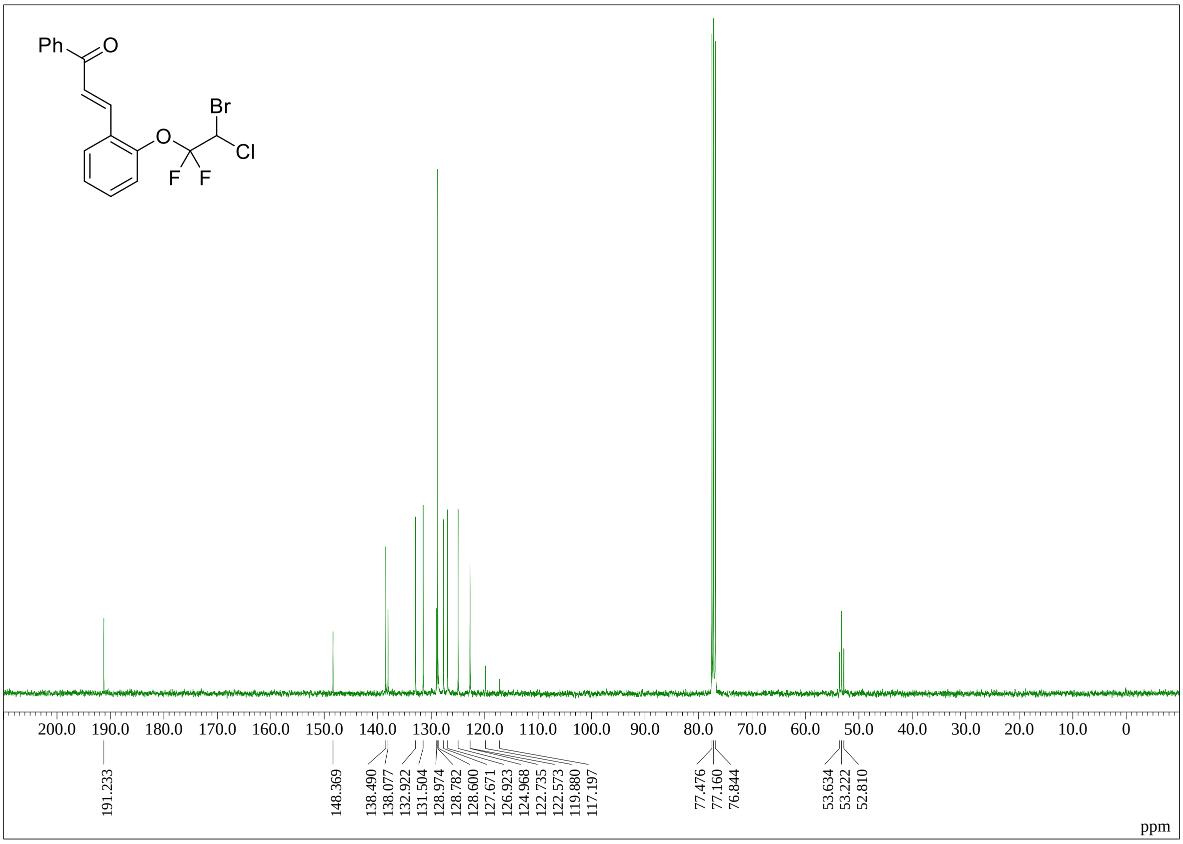
Ph
O
Br
O
Cl
F F
200.0 190.0 180.0 170.0 160.0 150.0 140.0 130.0 120.0 110.0 100.0 90.0 80.0 70.0 60.0 50.0 40.0 30.0 20.0 10.0 0
191.233
148.369
138.490
138.077
132.922
131.504
128.974
128.782
128.600
127.671
126.923
124.968
122.735
122.573
119.880
117.197
77.476
77.160
76.844
53.634
53.222
52.810
ppm

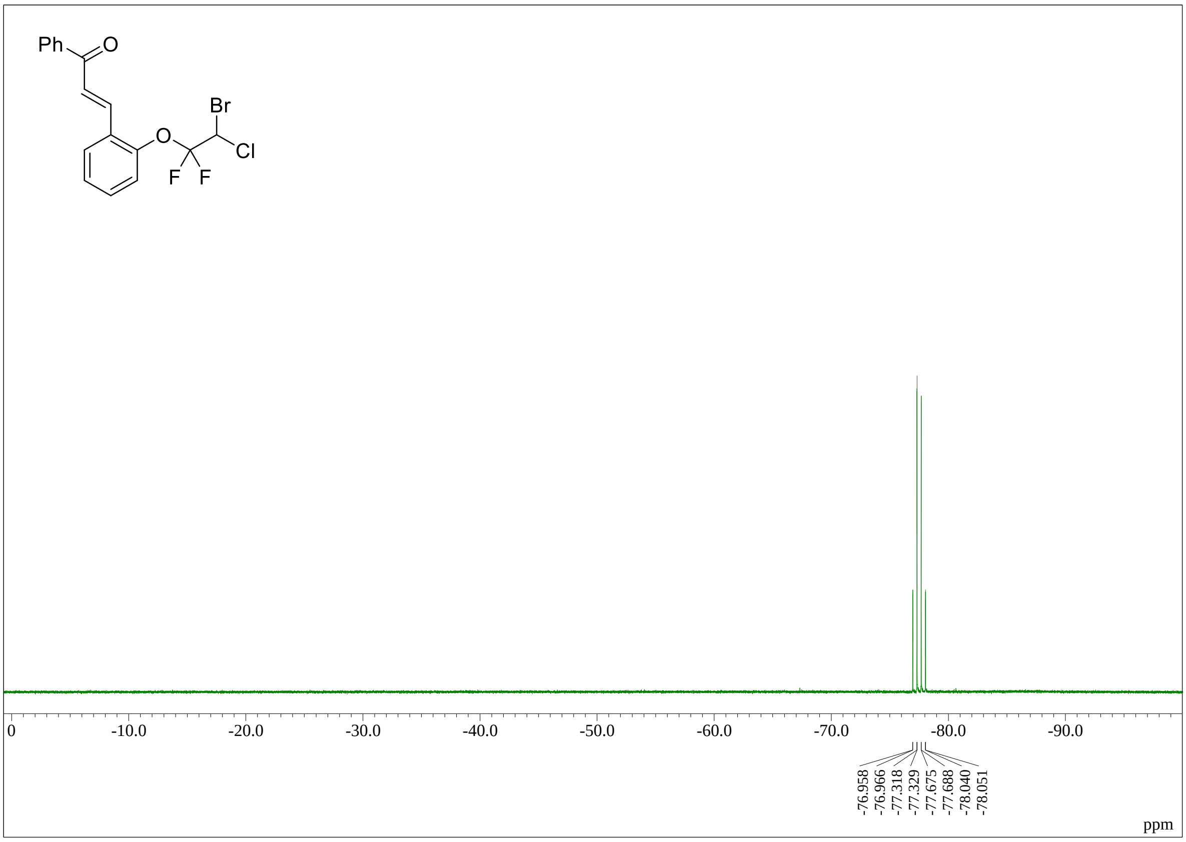
Ph
O
Br
O
Cl
F F
0 -10.0 -20.0 -30.0 -40.0 -50.0 -60.0 -70.0 -80.0 -90.0
-76.958
-76.966
-77.318
-77.329
-77.675
-77.688
-78.040
-78.051
ppm

o-Aminophenyl 2-bromo-2-chloro-1,1-difluoroethyl ether (2p)

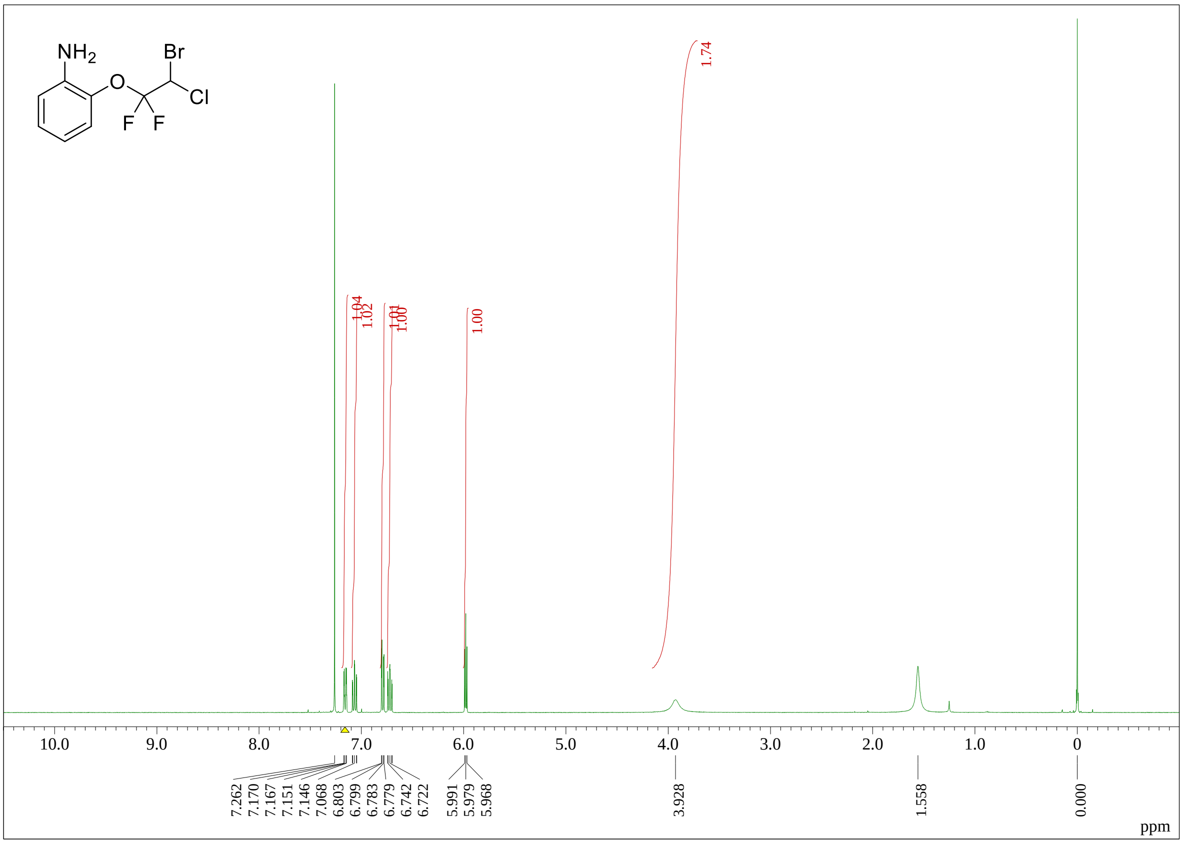

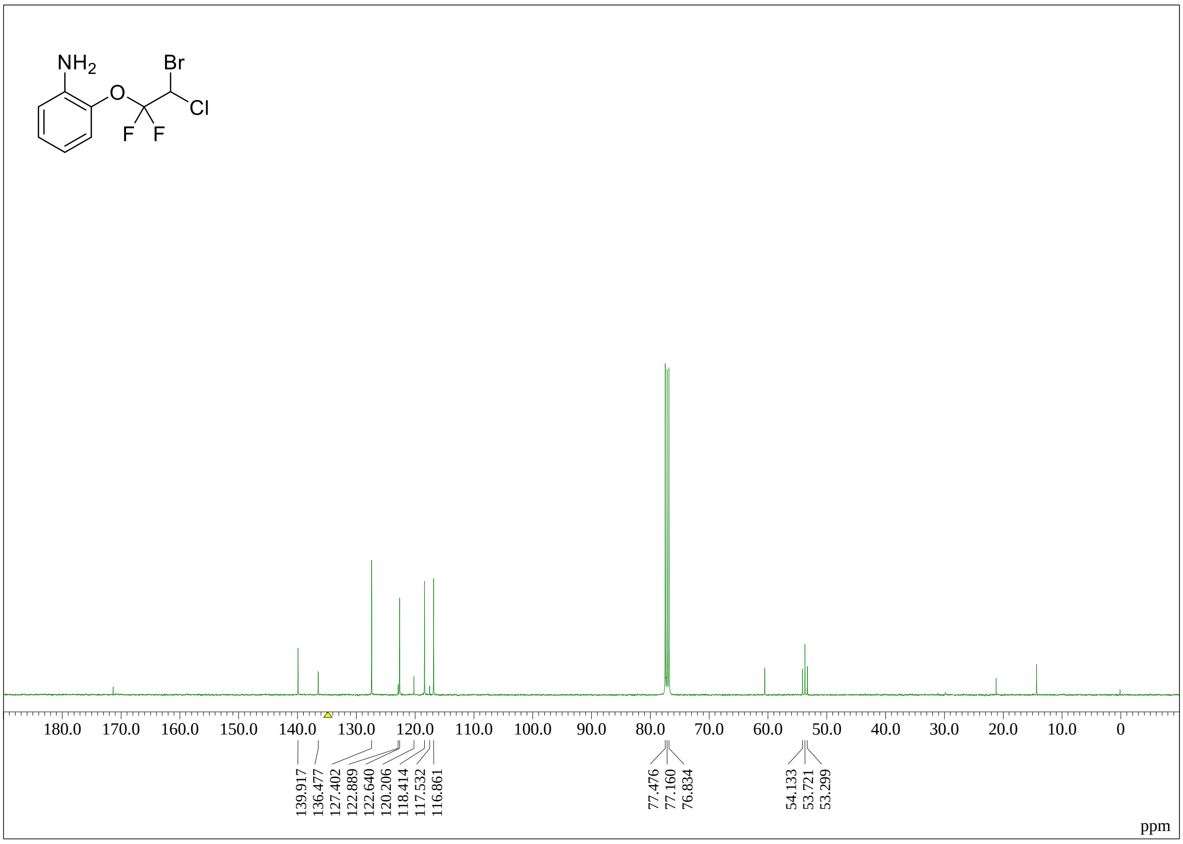

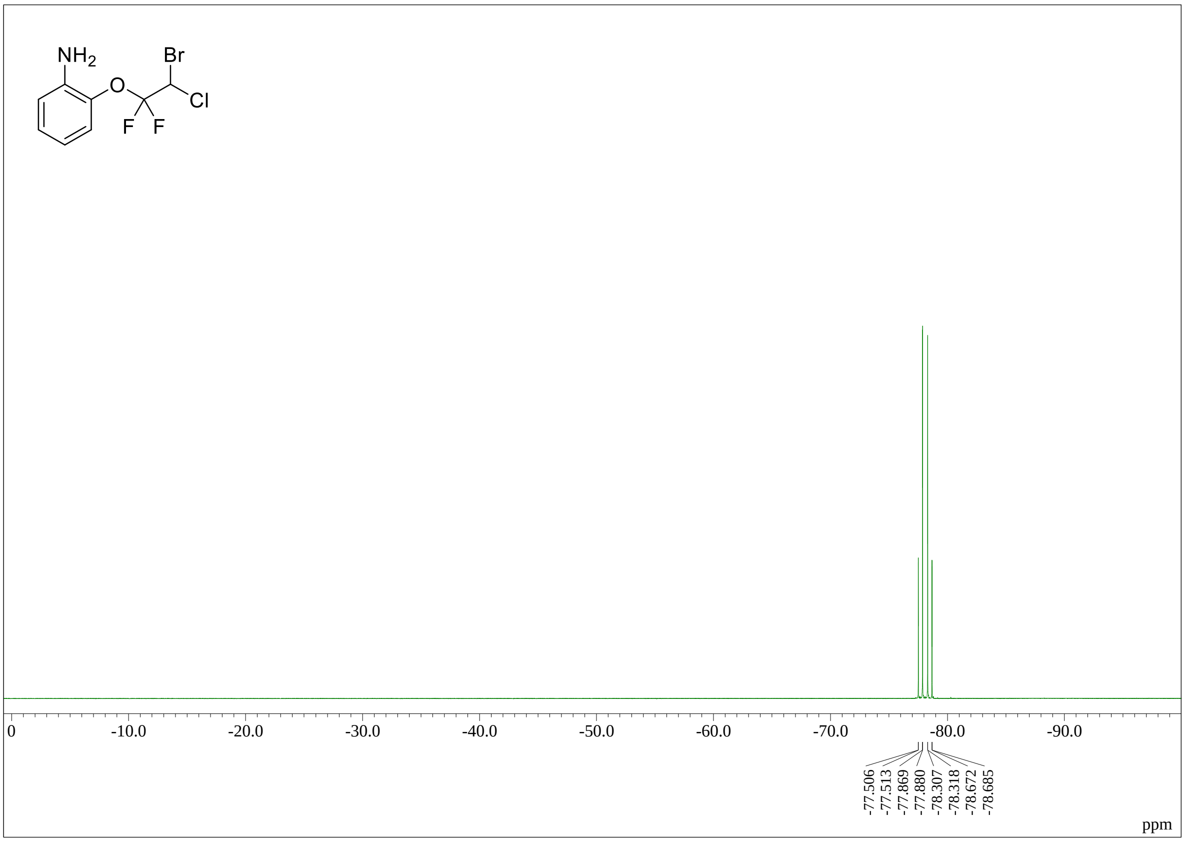

4-Benzoylmethyl-3-chloro-2,2-difluorochromane (3)

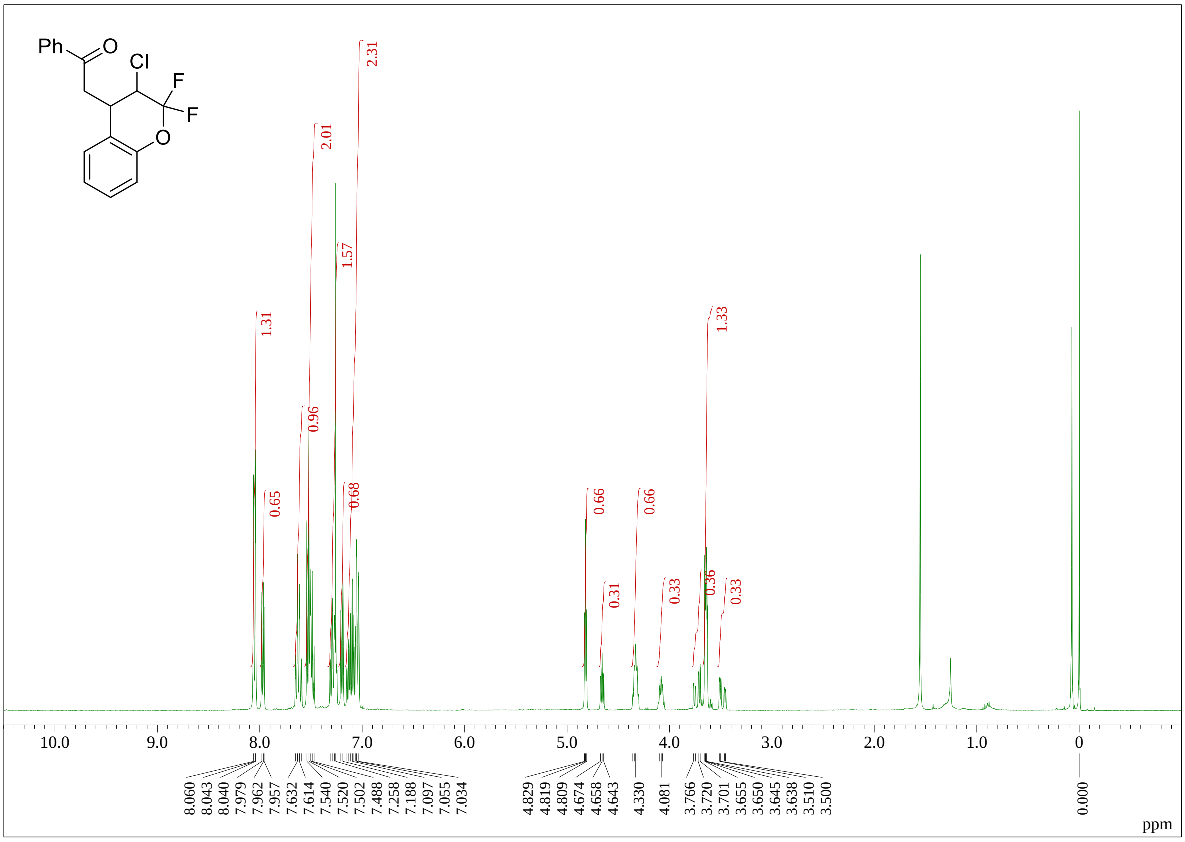

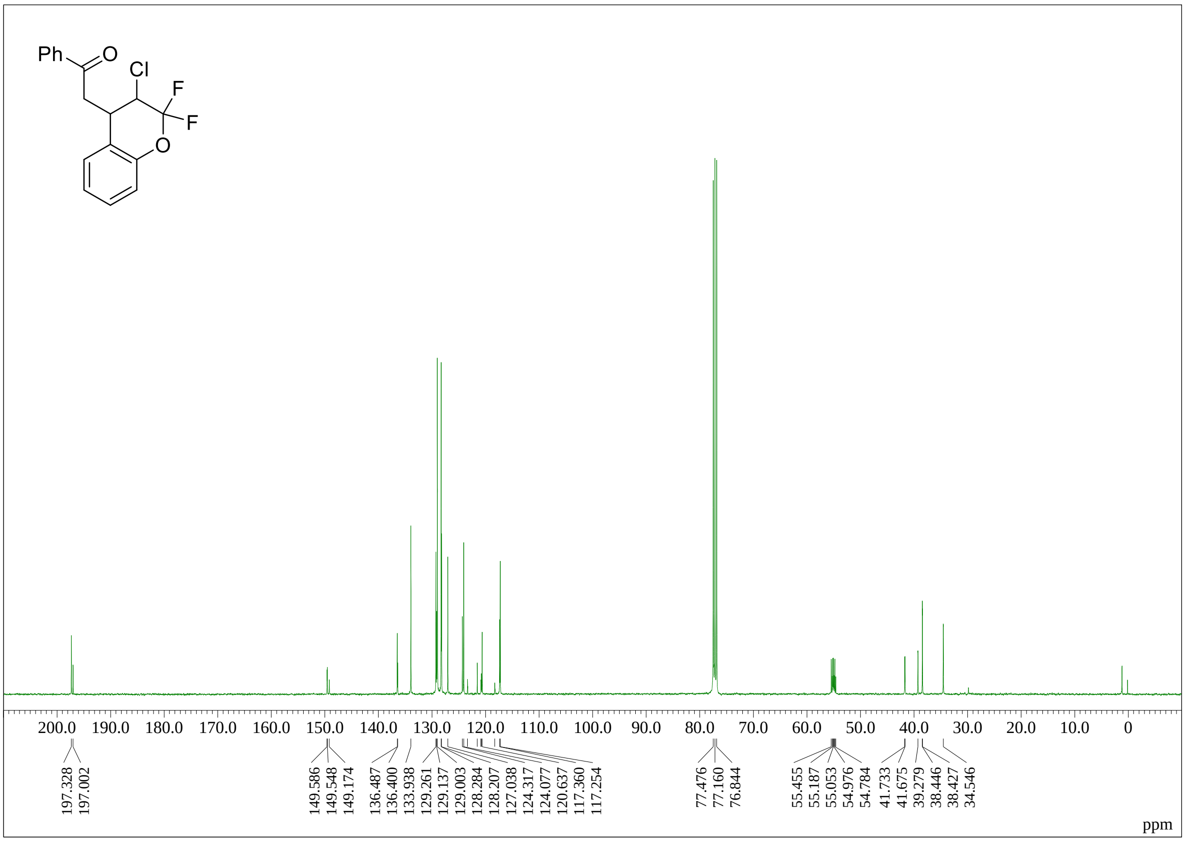
Ph
O
Cl
F
F
O
200.0 190.0 180.0 170.0 160.0 150.0 140.0 130.0 120.0 110.0 100.0 90.0 80.0 70.0 60.0 50.0 40.0 30.0 20.0 10.0 0
197.328
197.002
149.586
149.548
149.174
136.487
136.400
133.938
129.261
129.137
129.003
128.284
128.207
127.038
124.317
124.077
120.637
117.360
117.254
77.476
77.160
76.844
55.455
55.187
55.053
54.976
54.784
41.733
41.675
39.279
38.446
38.427
34.546
ppm

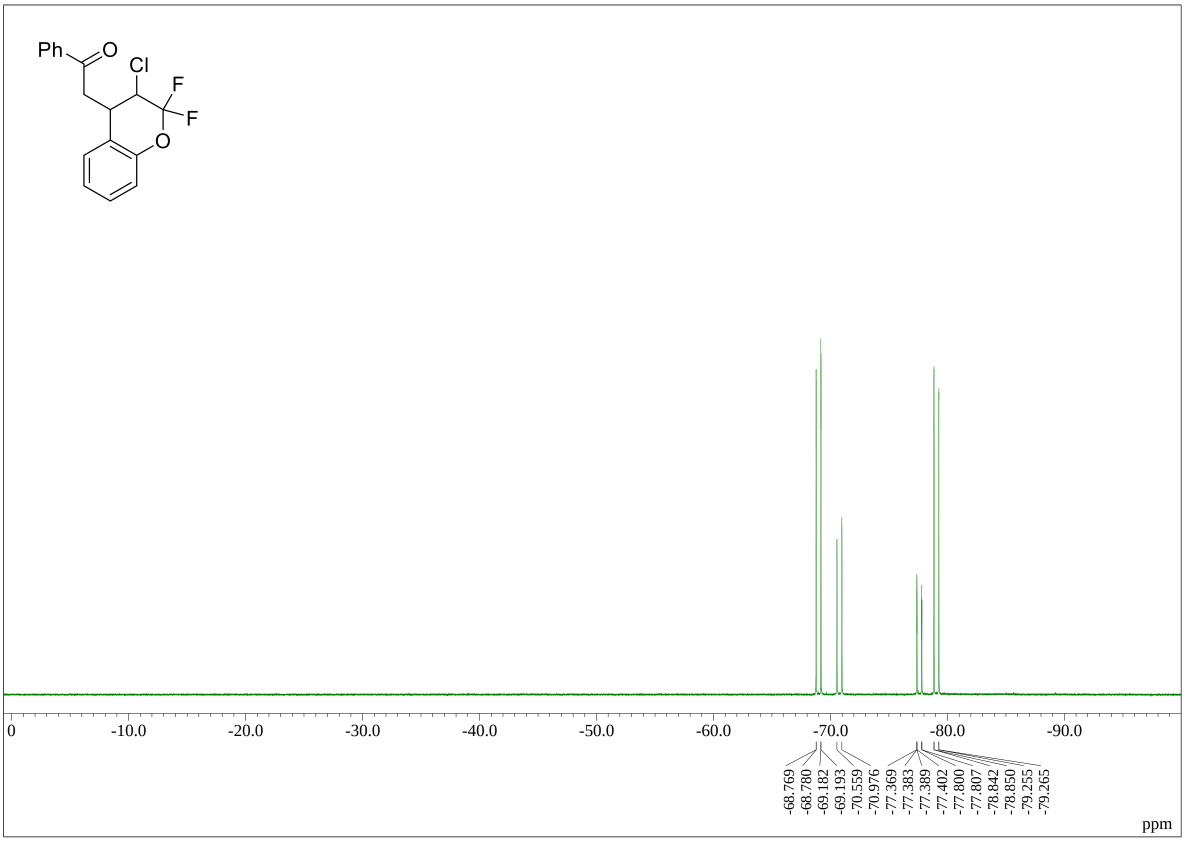
Ph
O
Cl
F
F
O
0 -10.0 -20.0 -30.0 -40.0 -50.0 -60.0 -70.0 -80.0 -90.0
-68.769
-68.780
-69.182
-69.193
-70.559
-70.976
-77.369
-77.383
-77.389
-77.402
-77.800
-77.807
-78.842
-78.850
-79.255
-79.265
ppm
